# Hepatocyte Ploidy Is a Diversity Factor for Liver Homeostasis

**DOI:** 10.3389/fphys.2017.00862

**Published:** 2017-10-31

**Authors:** Clemens Kreutz, Sabine MacNelly, Marie Follo, Astrid Wäldin, Petra Binninger-Lacour, Jens Timmer, María M. Bartolomé-Rodríguez

**Affiliations:** ^1^Faculty of Mathematics and Physics, Institute of Physics, University of Freiburg, Freiburg, Germany; ^2^Center for Systems Biology (ZBSA), University of Freiburg, Freiburg, Germany; ^3^Freiburg Center for Data Analysis and Modelling, University of Freiburg, Freiburg, Germany; ^4^Clinic for Internal Medicine II/Molecular Biology, Faculty of Medicine, Medical Center, University of Freiburg, Freiburg, Germany; ^5^Clinic for Internal Medicine I/Lighthouse Core Facility, Faculty of Medicine, Medical Center, University of Freiburg, Freiburg, Germany; ^6^BIOSS, Centre for Biological Signalling Studies, University of Freiburg, Freiburg, Germany

**Keywords:** hepatocytes, insulin, signaling, metabolism, polyploidy, liver

## Abstract

Polyploidy, the existence of cells containing more than one pair of chromosomes, is a well-known feature of mammalian hepatocytes. Polyploid hepatocytes are found either as cells with a single polyploid nucleus or as multinucleated cells with diploid or even polyploid nuclei. In this study, we evaluate the degree of polyploidy in the murine liver by accounting both DNA content and number of nuclei per cell. We demonstrate that mouse hepatocytes with diploid nuclei have distinct metabolic characteristics compared to cells with polyploid nuclei. In addition to strong differential gene expression, comprising metabolic as well as signaling compounds, we found a strongly decreased insulin binding of nuclear polyploid cells. Our observations were associated with nuclear ploidy but not with total ploidy within a cell. We therefore suggest ploidy of the nuclei as an new diversity factor of hepatocytes and hypothesize that hepatocytes with polyploid nuclei may have distinct biological functions than mono-nuclear ones. This diversity is independent from the well-known heterogeneity related to the cells' position along the porto-central liver-axis.

## Introduction

Somatic eukaryotic cells are usually diploid, i.e., have a pair (2n) for each set of n chromosomes. Cells may also possess greater than two sets of chromosomes, a condition which has been termed polyploidy. Such polyploid cells can either be mononuclear or binuclear. Polyploid cells with 4n in a single nucleus occur if karyokinesis has failed for a diploid cell, whereas two diploid nuclei (2^*^2n) emerge in the case of failure of cytokinesis. Since both events may occur repeatedly and can succeed each other, further combinations like 8n, or multinucleated cells with polyploidy nuclei, e.g., 2^*^4n, can occur.

Polyploidy was first identified more than a century ago and represents a universal biological phenomenon (Comai, 2005; Otto et al., 2012). By modulating gene expression in plants, polyploidy has been considered as an evolutionary adaptation to environmental changes (Masterson, 2011). Polyploidy of all somatic cells is uncommon in contemporary mammals (Svartman et al., 2005), presumably due to genomic incompatibility (Mable, 2004). However, genome duplication is considered a driving force in the early evolution of vertebrates (Panopoulou and Poustka, 2005), including primates (Bailey et al., 2002).

Adult mammals retain their capacity to generate polyploid cells under stress conditions such as wound healing (Ermis et al., 1998), hypertension (Vliegen et al., 1995), or after partial hepatectomy (Tamura et al., 1992). The aberrant polyploidy of cells arising during pathological conditions is considered to be a potential contributor to carcinogenesis. In fact, polyploid cells are found early in tumorigenesis and precede the development of aneuploid cells, i.e., cells with an intermediate level of DNA content (Storchova and Pellman, 2004; Ganem et al., 2007).

Only some mammalian tissues show a certain degree of polyploidy even under healthy conditions, e.g., the heart, skeletal muscle, and the liver (Carriere, 1967; Guidotti et al., 2003; Engel et al., 2006). Hepatocytes are usually diploid at birth and characteristically undergo dramatic changes during postnatal growth: diploid hepatocytes (2n) can either follow a normal cell cycle, or an adaptive cell cycle with incomplete cytokinesis, giving rise to binucleated diploid cells (2^*^2n) which seems to be triggered predominantly by TGFbeta1 (De Santis Puzzonia et al., 2016). Binucleated hepatocytes in turn can develop into polyploid mononucleated cells (4n) through failure of karyokinesis with fusion of the two separate spindles of both nuclei to form a single metaphase plate (Guidotti et al., 2003). The ploidy level of hepatocytes approaches a plateau several months postnatally and remains constant for life (Margall-Ducos et al., 2007). Any further changes of the total ploidy levels in the liver which emerge after this point will return to the original state within a few generations by polyploidization or polyploidy reversal (Duncan et al., 2010), thus suggesting that the maintenance of polyploidy status plays an important role in liver functionality. New multi-scale reconstruction techniques revealed unexpected zonation patterns of hepatocytes with different nucleation and DNA content in the liver tissue (Morales-Navarrete et al., 2015). It has also been shown that ploidy is increased during regeneration after partial hepatectomy although in this setting binuclear cells preferably build two mononuclear daughter cells (Miyaoka et al., 2012).

The degree and type of polyploidization (2^*^2n, 2^*^4n, 4n …) in the liver varies greatly between mammalian species (Anatskaya et al., 1994). For example, in the rat 80–90% of adult hepatocytes are polyploid (Styles, 1993), compared to 30–40% in humans. The percentage of polyploid hepatocytes seems to be regulated by hormones, with thyroid hormones playing a key role (Torres et al., 1999). In addition, insulin is known to trigger polyploidization shortly after birth by a program induced during the suckling-to-weaning transition phase (Celton-Morizur et al., 2009).

Polyploidization is not an obligatory characteristic for normal liver function in mammals. For example guinea pigs have very few binuclear hepatocytes (Styles et al., 1988) and in healthy woodchucks no polyploid hepatocytes have been found (Cullen et al., 1994). While the existence of polyploidy and binucleation in the liver are extensively described, their biological advantages are not yet defined or understood. While the polyploidy level of hepatocytes in many species is well-known, a detailed insight into the functional consequences of polyploidy with regard to binucleation is still lacking.

We have developed approaches to experimentally distinguish between cellular and nuclear ploidy in freshly isolated hepatocytes at the single cell level, and for separating cells according to their nuclear ploidy independent of the total cell ploidy. We found that the enzymatic activity, basal gene expression and the ability to bind insulin differ according to nuclear ploidy, but is almost independent from the total cell ploidy of the hepatocytes. More than 30 percent of genes were differentially expressed in hepatocytes when comparing cells with low and high affinity to insulin, indicating strong expression differences due to altered nuclear ploidy. We thereby provide the first evidence for a complex relationship between nuclear ploidy status of hepatocytes and heterogeneity of biological functions, which seems to be independent of their total ploidy and their position along the liver-sinusoid.

## Materials and methods

### Animals and common materials

Male C57BL6 mice of 8–16 weeks of age were obtained from the Charles River Laboratory (Sulzfeld, Germany). The institutional Animal Care and Use Committee at Freiburg University approved all of the procedures. Williams medium and FBS were obtained from Biochrom (Merck-Millipore, Berlin, Germany); dexamethasone was taken from Sigma (Sigma-Aldrich, Taufkirchen, Germany). For the presented results, a total of 54 experiments with each 2–3 mice (depending on the number of required cells for a specific assay) were performed. Details are provided in the Supplementary Table 5.

### Isolation and cultivation of hepatocytes

Two to three mice were used for the isolation of hepatocytes from the liver and pooled together for each experiment in order to have a good trade-off between reducing heterogeneity of hepatocytes from different mice and saving individuals. Hepatocytes were isolated and cultivated according to a standard operating procedure developed for studying a range of signaling pathways in hepatocytes under comparable conditions (Klingmüller et al., 2006). Briefly, after their isolation from the liver by treatment with collagenase, 3^*^10^7^–4^*^10^7^ hepatocytes per treated liver hepatocytes were seeded at a density of 10^5^/cm^2^ in rat collagen (BD, Germany) coated cell culture dishes and incubated with adhesion medium (Williams medium plus 10% FBS and 100 nM dexamethasone) for 4 h to ensure adherence to the dish. The exact number of cells used per dish was dependent of the surface and is summarized in the Supplementary Table 5. Cells were then thoroughly washed to eliminate dead cells with PBS and incubated with serum free medium containing dexamethasone for 20 h. Five hours prior to the experiments, the cells were washed again and incubated with serum free medium without dexamethasone

### Propidium iodide (PI) labeling

DNA content is assessed by labeling the DNA with Propidium Iodide (PI). Hepatocytes were fixed in 2% paraformaldehyde (Sigma-Aldrich), washed with PBS and incubated with 90% methanol (Sigma-Aldrich) on ice and analyzed 30 min after incubation with PI-solution (Sigma-Aldrich) by FC (FACSCalibur, BD Biosciences, Heidelberg, Germany).

### High-content screening (HCS)

Hepatocytes were cultured onto eight-well chambered μ slides (Ibidi, Martinsried, Germany) overnight. Overnight cultivation enables polarization of the cells, which is disturbed by collagenase treatment during isolation. For insulin-binding experiments, polarization is crucial since the receptor is only expressed on the sinusoidal side of the hepatocyte. Cell membranes were stained with AlexaFluor 546 labeled anti-ß-catenin antibodies (Cell Signaling, Frankfurt, Germany). One slide with eight independent chambers was used for any condition tested in each experiment. DNA-labeling was performed with 4′, 6-Diamidin-2-Phenylindol (DAPI, Sigma-Aldrich). Images were acquired at room temperature with an Olympus ScanR high content screening station (Olympus Europe, Hamburg, Germany), using a 20 x LUCPLFLN, N.A. 0.45 objective and ScanR acquisiton software (v.2.2.09). Fluorescence emission for DAPI was measured between 437 and 475 nm, green fluorescence (FITC and GFP) between 510 and 550 nm and AlexaFluor 546 between 573 and 613 nm. A total of 60–80 pictures were taken per chamber with a robot covering the whole slide.

### Quantitative analysis of HCS data

Analysis of fluorescence microscopy images was done using the Olympus ScanR analysis software (v.1.2.06). Hepatocytes were stained with DAPI and this staining was used for analysis. The main object mask was defined using an intensity threshold without use of a watershed algorithm, in order to keep nuclei from bi-nucleated cells within the same main object. Objects were divided into cells with double and single nuclei by gating on nuclear area vs. nuclear circularity. These gates were then combined with maximum DAPI Intensity vs. total DAPI intensity to form cell cycle profiles for both types of cells. Levels of viral expression after adenovirus infection and insulin bound to cells were both detected using subobject masks covering the entire cell, nucleus and cytoplasm, and based on the original main object mask using a DAPI threshold. All pictures obtained within an experiment were analyzed together independently on the treatment performed.

### Adenoviral infection

The magnitude of GFP expression after adenoviral infection (Soboleski et al., 2005) was used as as reporter for gene expression of the cells 24 h after infection which was augmented by functional analyses of differential gene expression. For this purpose, hepatocytes were infected 3 h after their isolation with an adenovirus encoding for green fluorescence protein (GFP, Adeno-easy technology, Qbiogene, MP Biomedicals Europe, Illkirch Cedex, France). The virus amount used is indicated in the results. On average, more than 95% of the cells were infected by the adenovirus.

### Carboxyfluorescein succinimidyl ester (CFSE) labeling

Hepatocytes were incubated directly after isolation for 10 min at 37°C with 10 μM Carboxyfluorescein Diacetate Succinimidyl Ester (CFDA-SE, Molecular Probes, Life Technologies, Darmstadt, Germany) and immediately fixed.

### Insulin incubation

Hepatocytes were incubated 24 h after isolation with human recombinant insulin covalently bound to fluorescein isothiocyanate (insulin-FITC, Sigma-Aldrich) in serum free medium for the times specified in the figures.

### Automatic, quantitative analysis of flow cytometry data

As described in more detail below, an automated processing of the raw experimental data obtained by flow cytometry has been established for this project in order to analyse hundreds of experiments/^*^.fcs data sets in a standardized and unbiased manner. This procedure comprises a so-called *2D-analysis* preprocessing step where a bivariate Gaussian mixture model was applied to automatically select viable hepatocytes based on forward- and side-scatter data. Then, a one-dimensional mixture model of two Gaussian distributions was used for the FITC channel to analyse the bimodal distribution of insulin binding (*1D-analysis*).

### Dynamics of insulin binding

A mathematical model based on ordinary differential equations was used to estimate rate constants for insulin binding, dissociation, and number of binding sites, as well as differences between cells with diploid and polyploid nuclei. Details are provided as Supplementary Material.

### Separation of hepatocytes with high and low insulin binding

Cells were washed and detached from the culture plate with trypsin after incubation with insulin-FITC. The single cell suspension was sorted according to the cells' insulin-FITC levels using a Beckman Coulter MoFlo legacy cell sorter with a 100 μm nozzle (Beckman Coulter, Krefeld, Germany).

### RNA extraction

Cells were lysed immediately after sorting using the AllPrep DNA/RNA/Protein Mini (Qiagen, Hilden, Germany). RNA was eluted from the RNA-binding membrane in nuclease-free water. RNA quality was examined using a RNA 2100 Bioanalyzer (Agilent Technologies, Böblingen, Germany).

### Affymetrix microarrays

Gene expression profiling was performed using the Affymetrix GeneChip Mouse Gene 2.0 ST Array (Affymetrix Europe, Wooburn Green, UK). All procedures, including *in vitro* transcription, labeling, hybridization, and detection were carried out as described in the Affymetrix GeneChip protocols (Gene-Chip expression analysis technical manual, 2012).

Data obtained by Affymetrix microarrays were pre-processed using the RMA Robust Multi-Array Analysis. Then, a linear model and the t-statistic was used to test for significantly regulated genes between the groups of hepatocytes, as well as for estimation of the fold-change and adjusting for differences between different preparations. Supplementary Figure 1 shows the distribution of the *p*-values assessing the significance of expression differences between the two cell entities with low and high amounts of insulin binding. Since we could show that the magnitude of insulin binding is strongly related to nuclear ploidy, we used insulin binding as a surrogate for nuclear ploidy. The gene-set regulation index (GSRI) (Bartholomé et al., 2009) was applied to estimate the percentage of regulated genes between both cell entities within functionally related groups of genes. For investigating up- and downregulation of gene ontology (GO) categories, the genes were first (independently of significance) subdivided into two subsets with positive or negative sign of the observed gene expression differences. Then, the GSRI was used to investigate significance by estimating the fraction of significantly regulated genes in each GO-category.

The MIAME-compliant microarray data can be found under the following link: http://seek.virtual-liver.de/data_files/3228?code=wK65y0lN4T5SRESZcfVYNCj374GPob%2FDXHPRIuEN.

### Statistics

Data are presented as mean ± SEM. Statistical significance of two-group comparisons were tested using Student's *t*-test. Differences were considered to be significant if *p* < 0.01. The statistical procedure for establishing a mathematical model for the dynamics of insulin binding, as well as for estimation of the parameters and confidence intervals, is summarized in the Supplementary Material.

## Results

### More than 75% of hepatocytes are polyploid containing diploid and polyploid nuclei with over 55% binuclear cells

**Figure 1:**
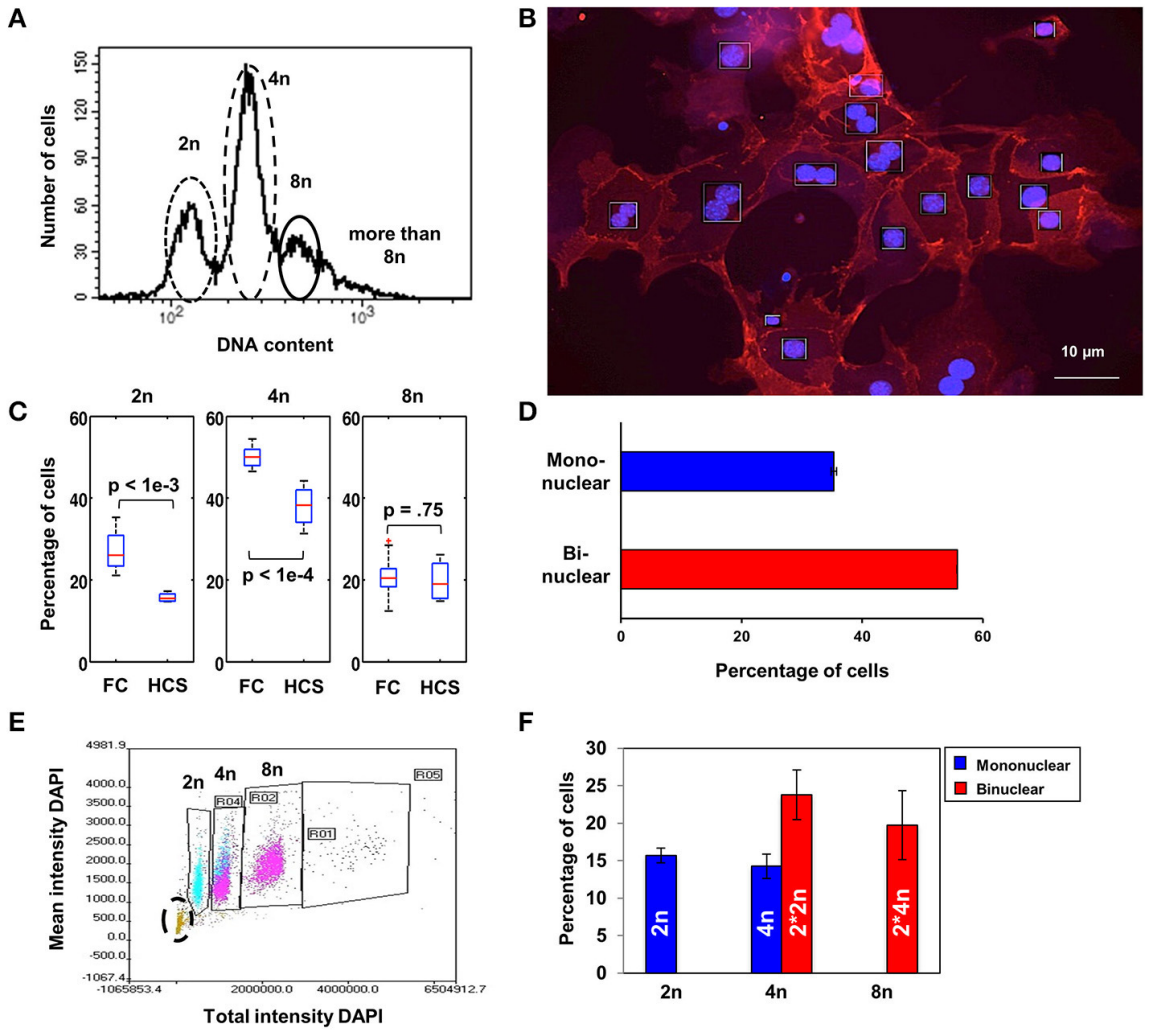
Diploid and polyploid nuclei are equally distributed in hepatocytes with binuclear cells representing the major population. **(A)** Separation of freshly isolated hepatocytes according to their DNA content by flow cytometry using PI. **(B)** Representative microscopy image used for the analysis of number of nuclei and amount of DNA per cell by High Content Screening (HCS) in hepatocytes after overnight culture and then staining with anti-ß-catenin and DAPI to determine the amount of DNA relative to the number of nuclei per cell. **(C)** Comparison of 2n, 4n, and 8n cells analyzed immediately after isolation or after overnight cultivation. There is a significant decrease (*t*-test) in the 2n and 4n populations after overnight incubation (HCS) compared to freshly isolated cells (FC). **(D)** Binuclear hepatocytes are the major population (56%) existing in the liver parenchyma as shown by HCS. **(E)** Quantification of hepatocytes under correlation with their nuclei number by HCS (those with one nucleus are displayed in blue and those with two nuclei in magenta). Apoptotic cells with very low DAPI intensity (lower left corner, in green highlighted by a black circle) have been excluded from the analysis. **(F)** Percentage of diploid (2n) and polyploid (4n) hepatocytes in the mono- and binuclear hepatocytes subset after quantitative analysis by HCS. Error bars represent SEM. The dead cells indicated in **(E)** by the black circle are excluded in **(F)**.

The DNA content of mouse hepatocytes directly after isolation has been assessed using Propidium Iodide (PI) labeling and flow cytometry. The subsets of cells with 2n, 4n, and 8n DNA contents are shown for one preparation in Figure 1A. In this example, mononuclear diploid hepatocytes (2n) make up around 25% of the cells, while the majority of cells (75%) are polyploid with at least 4n DNA content (55%, distributed in a single polyploid nucleus or two diploid nuclei), or hepatocytes with a higher DNA content (8n), representing binuclear 4n cells (20%). A quantitative analysis of 10 different cell preparations yielded 27.33 ± 1.45% cells with 2n, 50.09 ± 0.76% cells with 4n, and 20.72 ± 1.55% cells with 8n.

In order to differentiate between binuclear diploid and mononuclear 4n polyploid hepatocytes, cells were fixed after overnight culture to allow cell adherence and repolarization and stained with DAPI at saturating concentration to visualize the nuclei and ß-catenin was labeled with AlexaFluor 546 for visualizing the membrane by High-Content Screening (HCS, Figure 1B). For photodocumentation and quantitative analysis, 60 to 80 pictures per well were taken in each experiment. Compared to flow cytometry analysis performed directly after cell isolation, the percentage of viable hepatocytes in the 2n and 4n populations significantly diminish during overnight culture, whereas the 8n population only marginally change, as indicated by the boxplots in Figure 1C. The red line in the figure indicates the median, the blue box denotes the interquartile range and the black lines show the range of all measurements. The red cross indicates an outlier as commonly defined for boxplots.

HCS allows the additional identification and quantitation of mono- and binuclear hepatocytes, based on nuclear area and circularity, and in fact over 55% of the hepatocytes were binuclear (Figure 1D, representing the analysis of 320 serial images from eight culture dishes). By separating the cells based on total and mean DAPI intensity and additionally using the standard deviation of the DAPI staining, both the number of nuclei per cell and the amount of DNA per nucleus can be determined simultaneously (one nucleus blue, two magenta; Figure 1E). Apoptotic hepatocytes arising during overnight incubation (20%) were identified based on low DAPI intensity (Figure 1E, lower left corner) and correspond to the decrease in the number of cells in the 2n and 4n population after overnight cultivation as shown in Figure 1C. Taken together, hepatocyte cultures contained (average ± *SD*) 15.69 ± 0.86% mononuclear diploid cells (“2n”), 23.78 ± 3.30% binuclear diploid cells (“2^*^2n”), 14.30 ± 1.61% mononuclear polyploid cells (“4n”) and 19.75 ± 4.58% binuclear polyploid cells (“2^*^4n”) as shown in Figure 1F.

### Basal gene expression and enzymatic activity of hepatocytes depend on nuclear ploidy and not on total cell-ploidy

**Figure 2:**
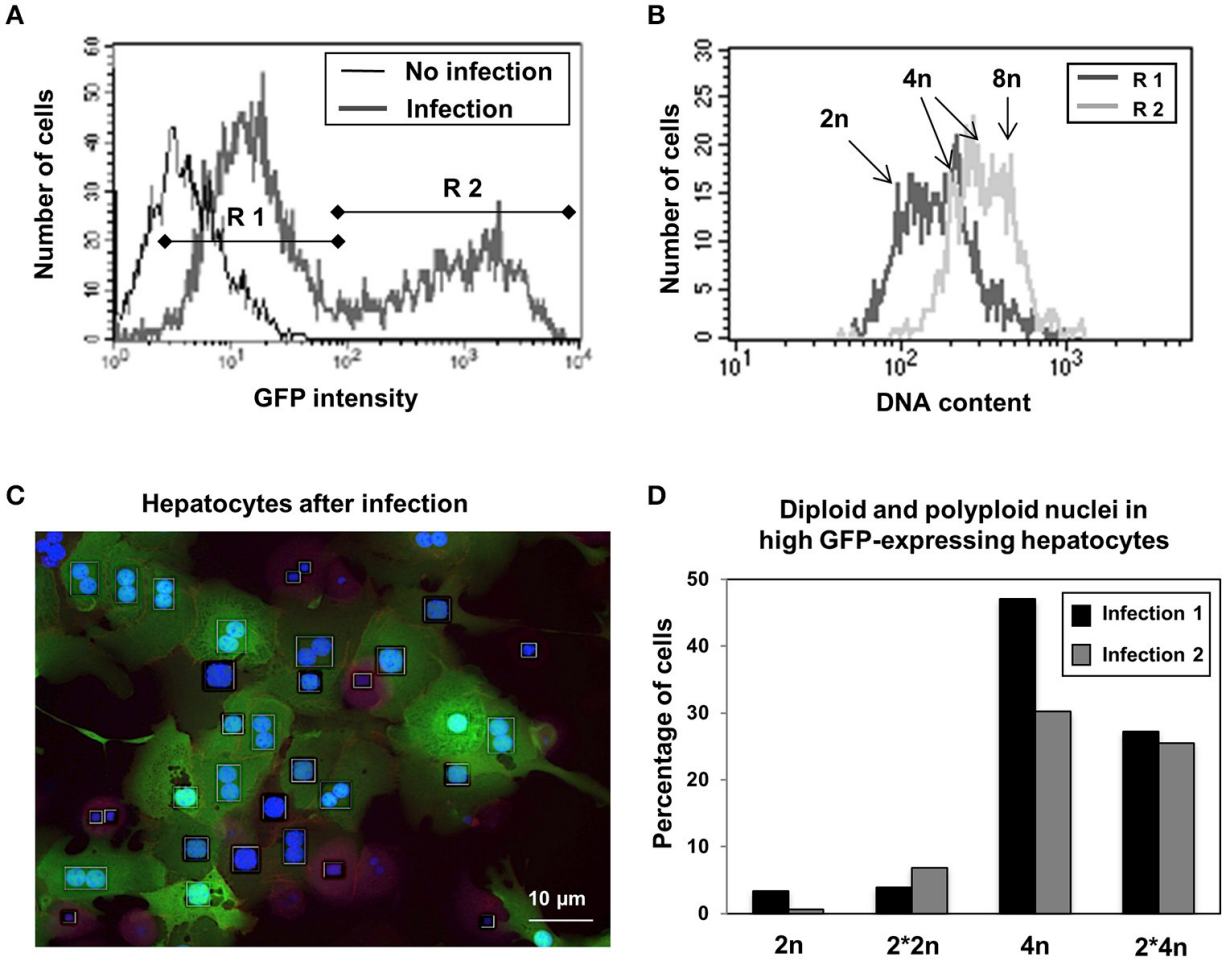
Hepatocytes containing polyploid nuclei have higher basal metabolism as indicated by Adenoviral protein expression. **(A)** Two cell populations could be detected based on differing levels of expression of GFP under the CMV promoter 24 h after adenoviral infection by flow cytometry. **(B)** Correlation between DNA amount and protein expression in low (R1) and high (R2) GFP expressing hepatocytes as seen by flow cytometry. **(C)** Representative microscopy image of GFP expressing hepatocytes after adenoviral infection after staining with DAPI. **(D)** The majority of the high GFP producing hepatocytes have polyploid nuclei, independent of the number of nuclei per cell. Percentage of diploid (2n) and polyploid (4n) nuclei containing cells in high GFP expressing hepatocytes in two independent cell preparations as analyzed by HCS.

GFP-Expression under the CMV promotor is a widely used tool to quantitatively visualize gene expression in individual eukaryotic cells. Since the intensity of GFP fluorescence is directly proportional to the mRNA abundance in the cells, the basal gene expression contributing to the total metabolic flux of the cells was assayed by quantifying the expression of GFP. Two hepatocyte populations (R1 and R2), with around than 100-fold different levels of GFP expression could be identified by flow cytometry 24 h after infection (Figure 2A). Although the number of viruses per cell also contribute to cell-cell variability, additional analyses shown in Figure 2B using PI for labeling DNA reveal that hepatocytes showing high levels of GFP (R2) are found in subsets of cells with intermediate to high levels of DNA (4n and 8n), whereas hepatocytes containing low levels of GFP are found in cells containing low to intermediate levels of DNA (2n to 4n cells, R1, Figure 2B). Figure 2C indicates a correlation of DNA levels and GFP expression. Detailed analysis of infected hepatocytes by HCS in order to separate the 4n population in mononuclear and binuclear cells showed that only hepatocytes with polyploid nuclei (4n or 2^*^4n) are able to express high levels of GFP, whereas polyploid hepatocytes containing two diploid nuclei express similarly low GFP-levels as do diploid mononuclear hepatocytes (Figure 2D). These results indicate that polyploid nuclei containing hepatocytes exhibit higher basal expression of the CMV promoter which might indicate an increased metabolic turnover compared to diploid cells, independent of the total cell ploidy.

**Figure 3:**
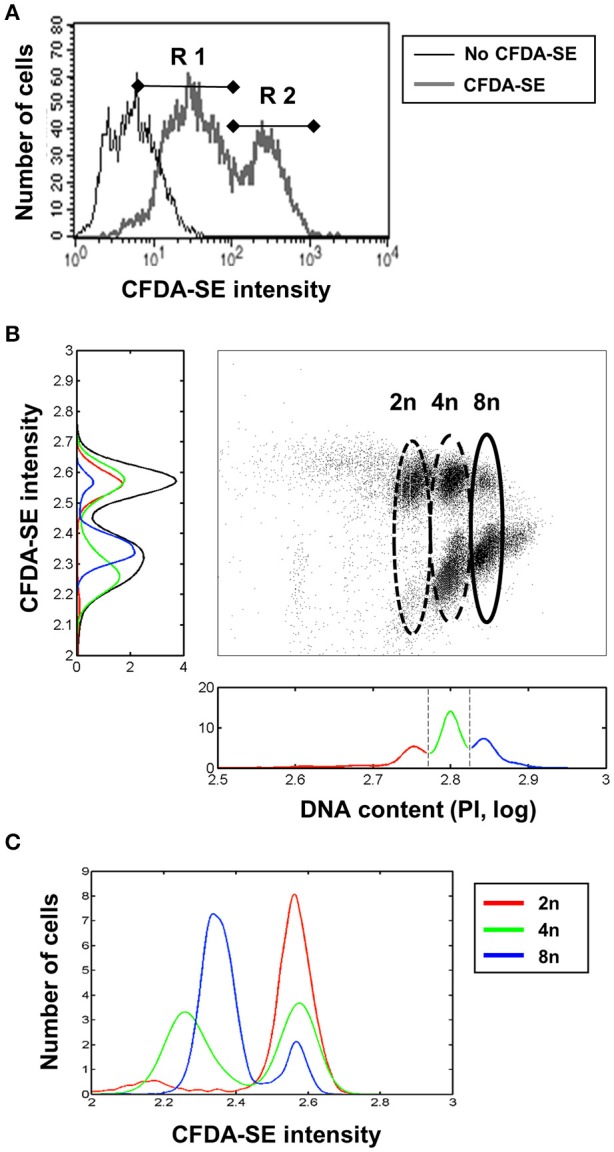
Higher substrate-induced metabolism in hepatocytes with diploid nuclei. **(A)** Two types of hepatocytes differing in their levels of CFDA-SE metabolism directly after isolation, as detected by flow cytometry. **(B)** 2n cells convert more CFDA-SE than 8n. Two different populations could be observed in cells containing intermediate amounts of DNA (4n). **(C)** Normalized CFDA-SE intensities in 2n, 4n, and 8n populations shown separately. The 4n cells could be separated into two different populations, probably dependent on the number of nuclei per cell (2^*^2n or 1^*^4n).

As a further indicator we quantified the levels of substrate-induced enzymatic activity by measuring esterase activity, which is a general indicator of substrate-induced cellular metabolism. For the measurement of intracellular esterase activity, the highly membrane permeant Carboxyfluorescein diacetate succinimidyl ester (CFDA-SE) was used. CFDA-SE possesses a rapid flux across the plasma membrane. Once in the cell esterases cleave the acetates from CFDA, the levels of fluorescent CFSE increase, which is much less permeable and binds to intracellular proteins, staining the cell. The amount of CFSE between cells primarily depends on the esterase activity within the cell. Directly after isolation from the liver, we measured the amount of CFSE which had accumulated in the hepatocyte 10 min after addition of 10 μM CFDA-SE. Flow cytometry shows two populations of hepatocytes, R1 and R2, which differ by their substrate-induced enzymatic activity, with R2 showing levels of CFSE 10 times higher than that of R1 (Figure 3A).

Comparison of esterase activity with DNA amounts by flow cytometry showed that the 2n hepatocytes exhibited high esterase activity, the 4n cells were split into two populations with either high or low activity, presumably corresponding to the 2^*^2n and 4n cells, respectively, and that the majority of the 8n cells exhibit low levels of esterase activity (Figure 3B). Although we could not directly analyse the number of nuclei present per cell, when considering the previously presented results on the frequency and behavior of mono- and binuclear hepatocytes, the two large clouds in Figure 3B seem to correspond to mono- and binuclear cells. Figure 3C shows the CFDA-SE intensity distribution of 2n, 4n, and 8n hepatocytes normalized to an area equal to one, demonstrating the existence of two CFDA-SE entities of 4n hepatocytes.

### Hepatocytes with diploid and polyploid nuclei differ in their affinity to insulin

**Figure 4:**
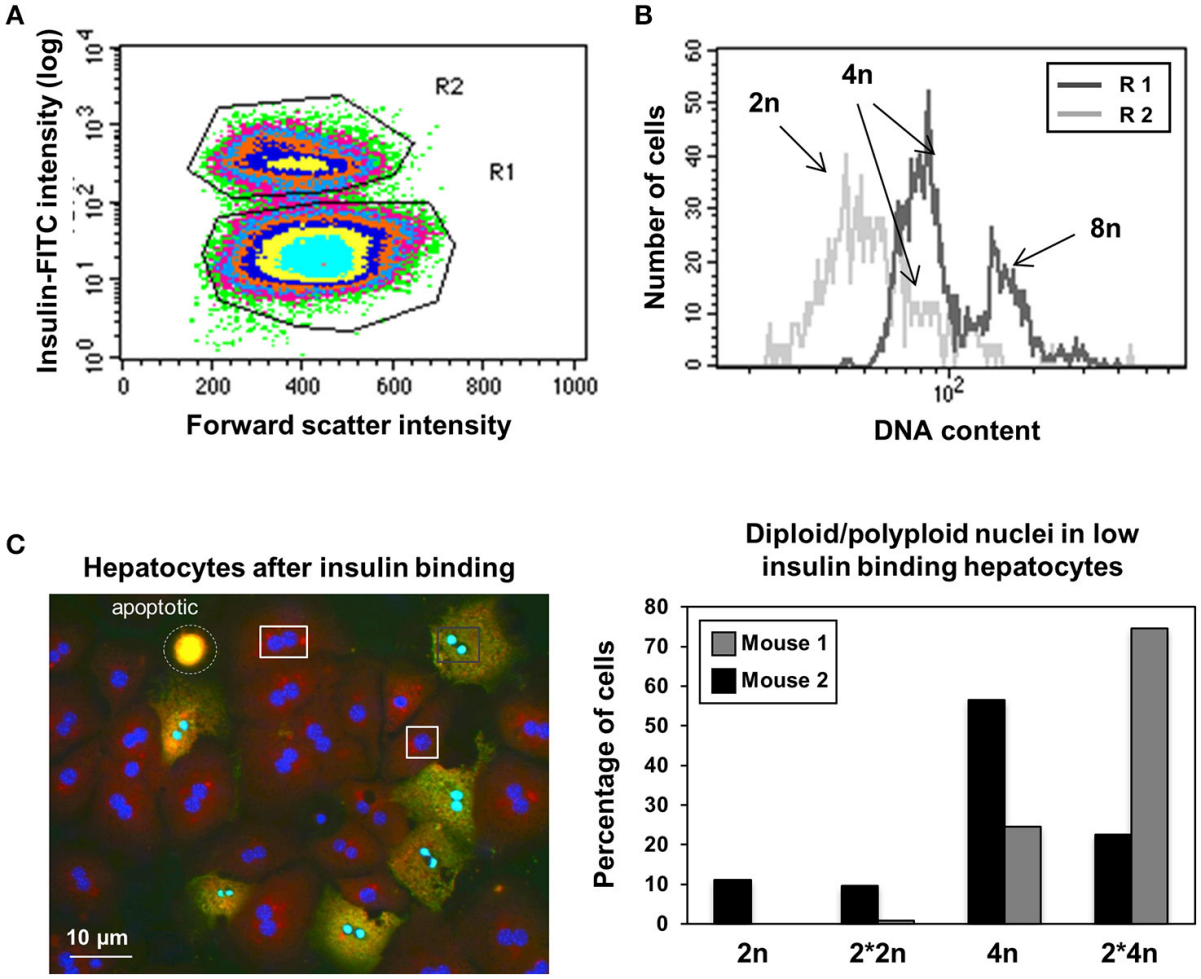
Diploid nuclei containing hepatocytes exhibit higher affinity for insulin. **(A)** Two types of hepatocytes observed according to their magnitude of insulin-binding exhibit similar cell-size in flow cytometry. **(B)** Flow cytometry analysis using insulin-FITC show higher insulin binding affinity in 2n cells compared to 8n cells. Again, two types of cells are visible in the 4n DNA containing cells. **(C)** Low affinity for insulin could mainly be observed in polyploid nuclei. Left: representative microscopy image shows cells with two nuclei in both the high binding and low binding subsets. Right: percentage of 2n and 4n nuclei in insulin low-binding cells as analyzed by HCS. Low binding cells contain polyploidy nuclei, independent of the number of nuclei per cell.

Insulin is a primary hormone affecting many metabolic functions in the liver. Insulin binding was analyzed by flow cytometry 15 min after its addition (10 μM) to hepatocytes which had been cultured overnight, again demonstrating the existence of two hepatocyte subpopulations with an ~10-fold difference in their capacity to bind insulin. There is no obvious correlation between binding and cell size as shown in Figure 4A. Consistent with the levels of esterase activity (Figure 3), high insulin binding was associated with low to intermediate DNA content (2n and 4n cells, R2 in Figure 4B), while hepatocytes with a low affinity for insulin have an intermediate to high DNA content (4n and 8n cells, R1 in Figure 4B). Among the 4n cells we again discover two entities characterized by different insulin affinities, which were further analyzed by HCS. As shown in Figure 4C, low insulin binding was associated with polyploid nuclei, while high insulin binding was associated with mono- and binuclear diploid hepatocytes. So again the two entities exhibiting different insulin affinities coincide with ploidy of the nuclei.

### Comprehensive, application-specific pre-processing for robust, automatic, and statistically valid analysis of flow cytometry

**Figure 5:**
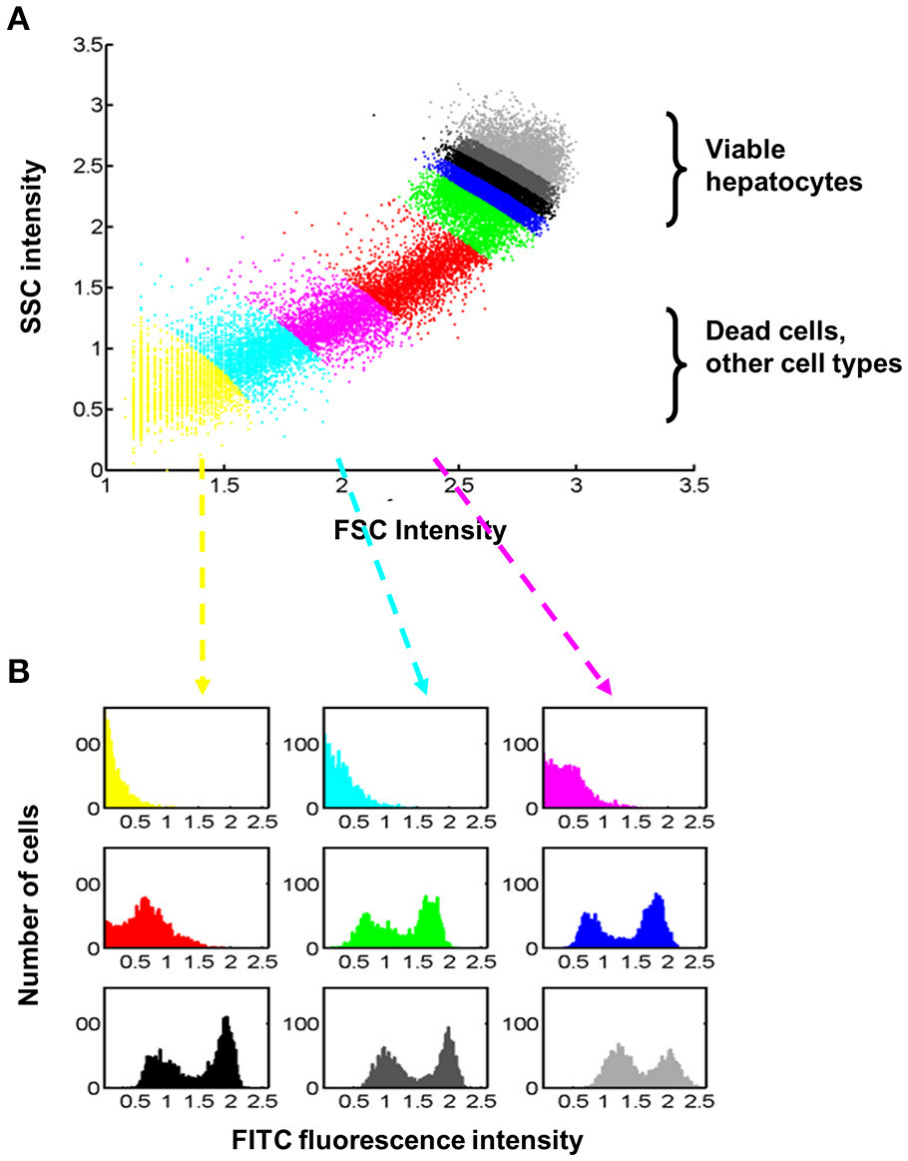
Relationship between selections of cells based on forward- and side-scatter intensities and the respective fluorescence intensity for insulin-FITC. **(A)** Dead cells, primarily originate from collagenase tissue digestion, and other cell types cannot be clearly separated from hepatocytes automatically. For illustration purpose, 9 different groups were defined containing the same number of cells but at different distances from the origin (FSC = 0, SSC = 0). We applied rather stringent thresholds for selection of viable hepatocytes and analyzed on average around 30.8% of cells. **(B)** The fluorescence intensity in the insulin-FITC channel is dependent on the selected forward- (FSC) and side-scatter (SSC) signals, which demand a statistically valid analysis which is robust against the definition of thresholds. The color of the histograms indicates the group defined in **(A)**. All viable hepatocytes (green, blue, black, and gray) show a bimodal distribution with regard to fluorescence (FITC).

An automatic data processing strategy was established for quantitatively evaluating the time- and dose dependency of insulin binding based on flow cytometry. As a first step, the cell population of interest, i.e., viable hepatocytes, has been selected based on signals in forward (FSC) and side scatter (SSC) in an initial pre-processing step, which is termed *2D-analysis* in the following experiment. Figure 5 illustrates that this selection step has an impact on the outcome in the insulin-FITC channel. For illustration purposes, 9 groups with equal numbers of events/cells were defined according to their distance from the origin (FSC = 0, SSC = 0) as shown in Figure 5A. The impact of the selection on the intensity distribution in the insulin-FITC channel is shown in Figure 5B. The colors of the histogram correspond to the group definition in Figure 5A. Although all viable hepatocytes show qualitatively the same, i.e., a bimodal, distribution, the quantitative outcome in terms of shape and location depends on the selection which was based on forward- and side scatter.

**Figure 6:**
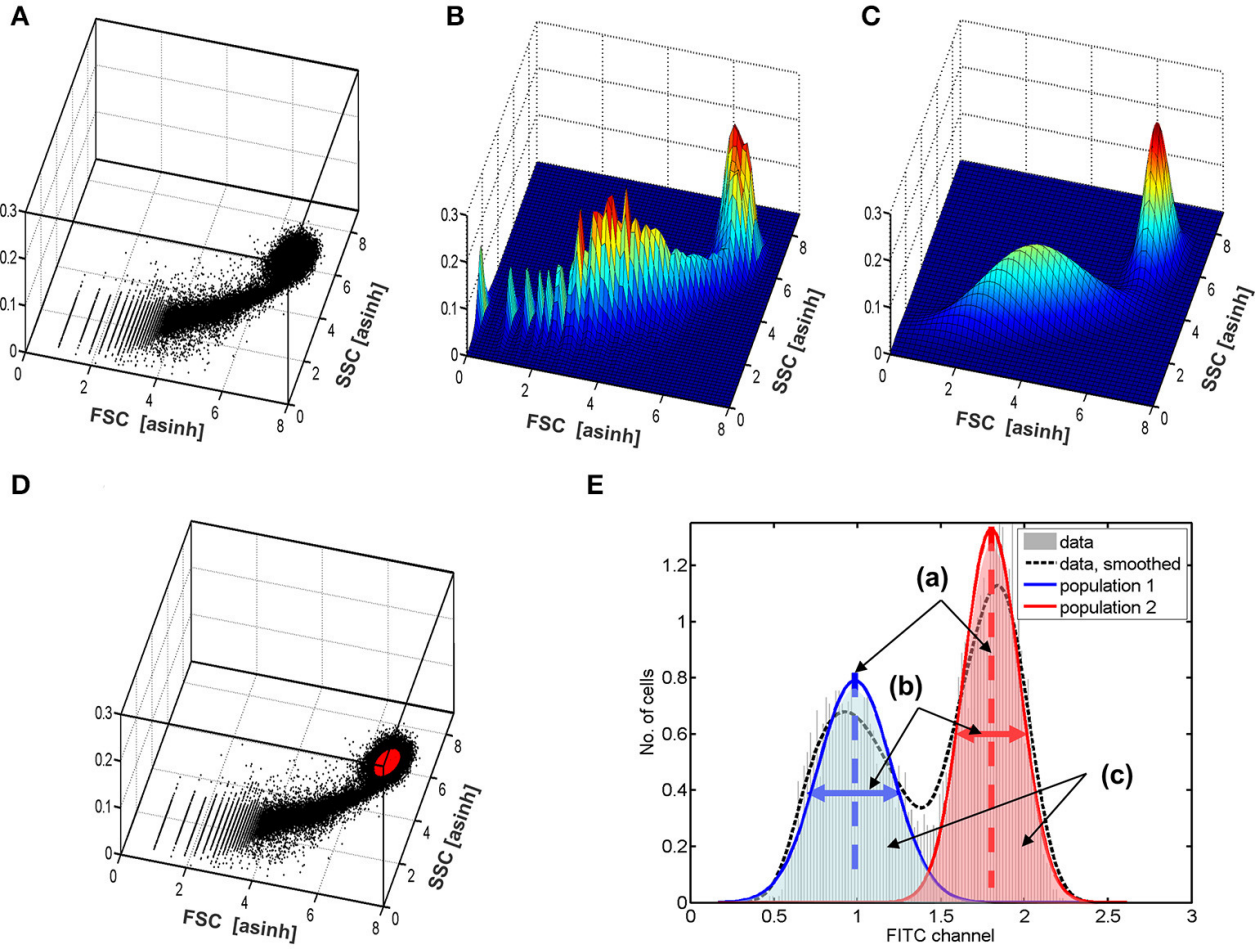
Demonstration of the data processing based on mixture models. Panel **(A)** shows the measurements as a scatterplot of forward vs. side-scatter signals. Panel **(B)** shows the same as a 2D histogram. The fitted Gaussian mixture model is plotted in **(C)**. Based on this fit, the cells are selected based on thresholds. One outcome, i.e., the selected cells are shown in **(D)** in red color. To obtain robust results, the outcomes are averaged over alternative reasonable data processing strategies using other data transformations (asinh and log) and thresholds. Panel **(E)** illustrates the obtained five numbers estimated for each data set from the histogram of the FITC channel: (a) two estimates of the average amount of insulin binding for the two groups of cells (dashed lines); (b) two variances for the two observed cell groups (horizonal arrows); (c) the proportion of cells in the two groups of cells, which can be calculated from the areas in both groups.

To ensure that the results in the fluorescence channel are robust against the implementation and settings chosen in the 2D-analysis, the outcomes were evaluated for several reasonable processing strategies: Three different transformations of the intensities were applied, namely the log-, asinh-, and boxcox transformations (Box and Cox, 1964). In addition, two different thresholds (a = 0.8 and a = 0.95) were evaluated with regard to the posterior probabilities for the class labels. Two further thresholds (b = 0.2 and b = 0.2) were used for posterior density. Figure 6 demonstrates the 2D-analysis which utilized a bivariate Gaussian mixture model for the selection of viable hepatocytes. Forward- and side scatter intensities obtained by flow cytometry were plotted as a scatterplot in Figure 6A and as a histogram in Figure 6B. Figure 6C shows the Gaussian mixture model fitted from the experimental data. The broad peak represents dead cells and other cell types, whereas the viable hepatocytes are located within the second, narrower peak. Based on such fitted mixture densities, the cells are further selected for analysis of the FITC channel by the two above introduced thresholds, a and b. An example of the cells which were finally selected is highlighted in Figure 6D in red. Figure 6E shows a bimodal distribution of the FITC fluorescence obtained for the viable hepatocytes selected.

Each FC data set was analyzed using all thresholds and transformations. The identified FITC channel of the identified viable cells was then further analyzed using a univariate, i.e., one-dimensional mixture-model of two Gaussian distributions to estimate the mean and standard deviation of both peaks in the bimodal distribution, as well as the proportion of cells belonging to each subtype as indicated in Figure 6E. The results were averaged over all 2D setups by a statistical model (Kreutz, 2011).

### Diploid and polyploid nuclei containing hepatocytes have similar kinetic shapes, albeit with different magnitudes

**Figure 7:**
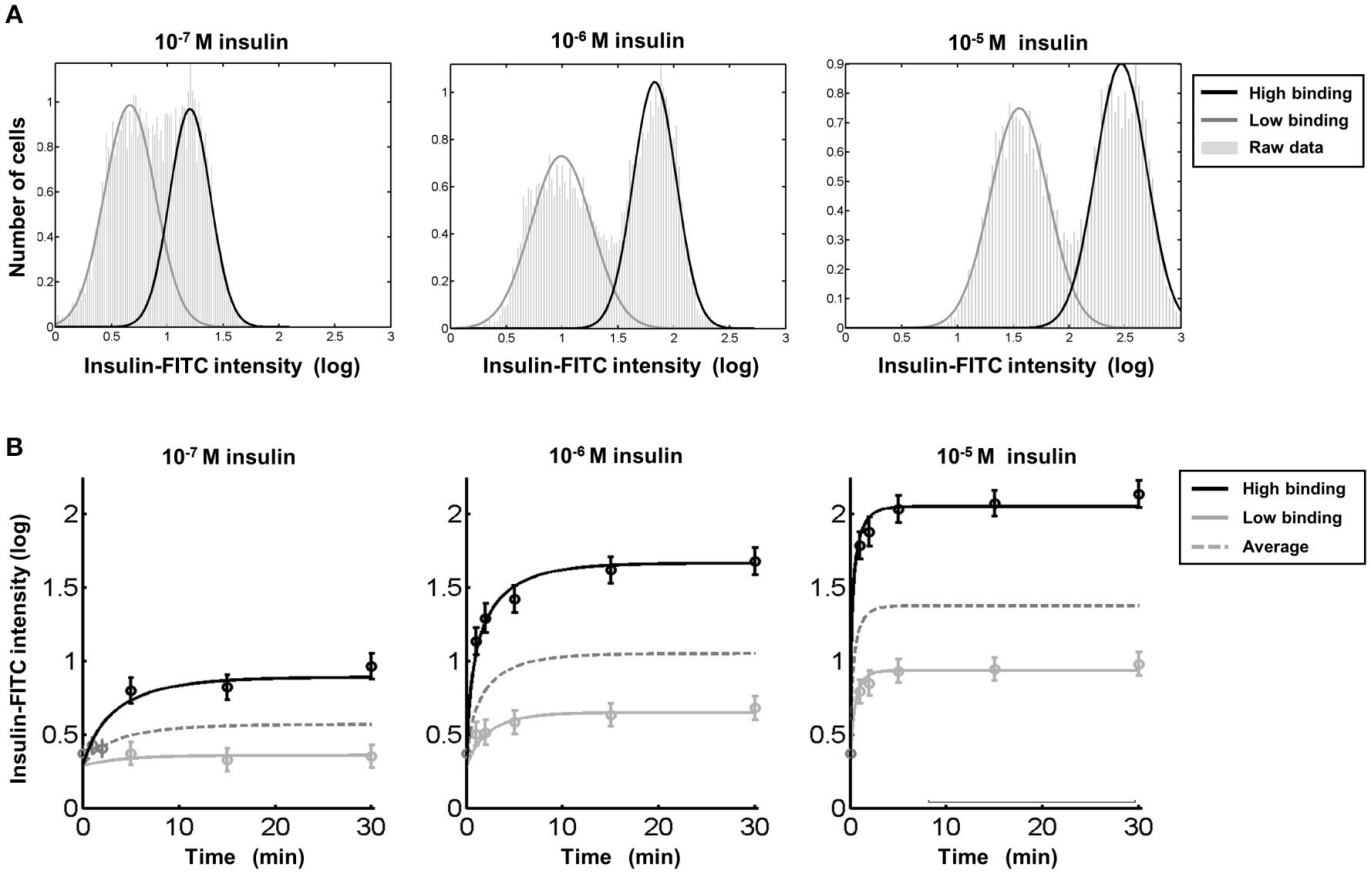
Insulin binding differs in hepatocytes with diploid and polyploid nuclei despite comparable time kinetics. **(A)** The low and high insulin binding hepatocytes could be found by flow cytometry at any insulin concentration tested. A mixture of two Gaussian densities is fitted to each data set to estimate the dose dependency in detail (Supplementary Information and Figures 5, 6). **(B)** The two entities arise immediately after the addition of insulin at all of the tested concentrations and have comparable saturating kinetics. The average shown as dashed lines represents insulin binding as it would be observed if the two types of hepatocytes could not be distinguished. For t = 0 as well as for the first three time points at the lower dose (left), the individual averages of both entities could not be calculated without a bias. Therefore, only the average over both entities is plotted and used for fitting of the kinetic model.

The comprehensive data analysis strategy described in the previous section was utilized for reliable estimation of the time and dose-dependency of insulin binding in both entities. The mean, i.e., the average amount of bound insulin, and the variance, i.e., cell-to-cell variability within both entities were determined for 196 flow cytometry datasets. The bimodal distribution of FITC-labeled insulin binding was seen throughout. Three representative data sets for three insulin concentrations evaluated 15 min after stimulation are shown in Figure 7A.

The dynamics of insulin binding were analyzed for three different insulin concentrations at six time points between 0 and 30 min. The average insulin binding intensity in each entity (mean of insulin binding within both cell populations) is depicted in Figure 7B. Both entities with low and high insulin binding can be clearly identified immediately after addition of insulin at all concentrations tested. Both hepatocyte subtypes exhibit rising dynamics up to dose-dependent steady state levels. The shape of the dynamics is similar for both cell entities, but the magnitude is increased for hepatocytes with diploid nuclei. Both cell-cell variability of insulin binding within each entity and the fraction of cells belonging to both hepatocyte subtypes was independent of the level of insulin exposure.

Next, we analyzed which mechanism of insulin binding could generate the observed difference between the two cell entities. For this purpose, a mathematical model describing the insulin binding kinetics using a mass action model was utilized to predict whether the difference originates from a difference in the number of binding sites, or rather from distinct complex formation- or dissociation rates. In the model there are two rate constants for the association of insulin to the receptors, for insulin dissociation, and for the number of binding sites per hepatocyte (Supplementary Text). Statistical analysis of the data indicated different parameters for the number of binding sites in both entities but no significantly different association/dissociation rates in the low and high insulin binding liver cells.

**Figure 8:**
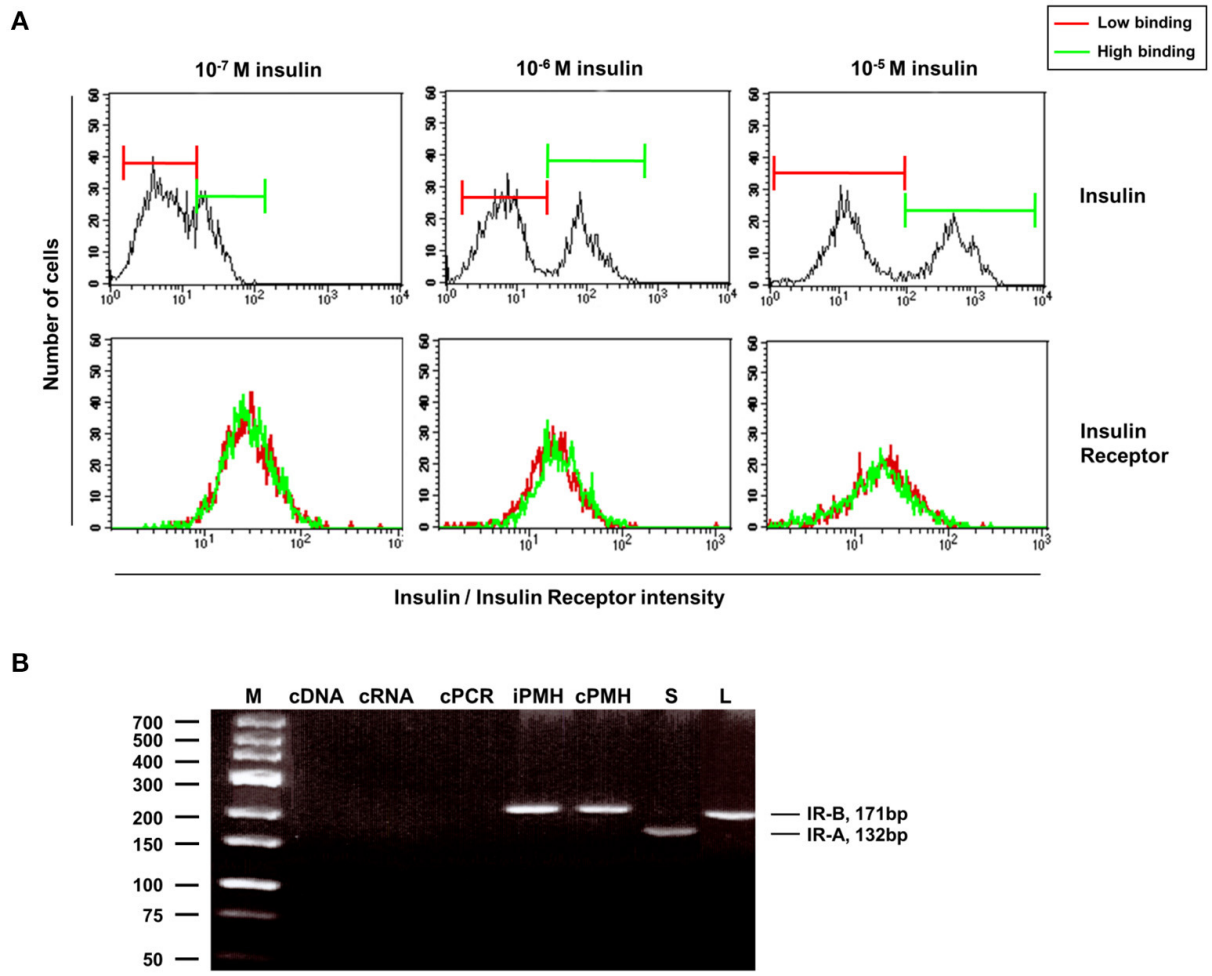
Similar receptor expression found in hepatocytes with low and high insulin binding as seen by flow cytometry. **(A)** Upper panel: separation of insulin-low binding and insulin-high binding hepatocytes by different doses of insulin 5 min after insulin incubation. Lower panel: similar IR expression in low and high insulin-binding hepatocytes after their separation according to their insulin affinity. **(B)** Expression of insulin receptor variants in primary cells as determined by RT-PCR using the fact that the variant B expresses an additional exon. GeneRuler Low Range DNA Ladder (Fermentas, Thermo Fisher Scientific, Darmstadt, Germany) was used as Marker (M). Controls were the following: PCR using water for the cDNA synthesis instead of RNA (cDNA); PCR using RNA as template instead the synthesed cDNA (cRNA); PCR using water in the PCR reaction instead cDNA (cPCR). iPMH, fresh isolated hepatocytes; cPMH, hepatocytes after overnight cultivation; S, spleen; L, murine liver.

In order to corroborate the outcome of the mathematical model, insulin receptor (IR) expression was analyzed in hepatocytes by flow cytometry 5 min after insulin incubation. Surprisingly, IR expression was similar between low and high insulin binding hepatocytes (Figure 8A). In addition, there was no difference in the expression of IR splicing variants between hepatocyte subtypes (Figure 8B). Using the fact that variant B of the receptor expresses an additional exon, the expression of insulin receptor variants in primary cells was determined by RT-PCR.

To validate the quantification of splice-variants of the insulin receptor, we analyzed cells derived from the spleen since IR-A is known to be expressed in the spleen. Indeed, expression of the IR-A splice variant could only be found in the spleen (S) but not in the murine liver (L). No changes in the variant expression could be shown in the freshly isolated hepatocytes (iPMH) or after overnight incubation (cPMH). Therefore, the observed low and high amounts of insulin binding are neither related to expression of the IR-A splice variant, nor to the expression of hybrid receptors consisting of IR-A/IR-B heterodimers. These results could not sustain the different number of binding sites predicted by the kinetic model. Therefore, a difference between message level and receptor abundance in the cells and/or at the surface has to be responsible and the insulin receptors have to be blocked or enhanced by alternative mechanisms like receptor clustering, translocation into membrane microdomains or intracellular compartments generating the differences observed between both entities.

### Diploid and polyploid nuclei containing hepatocytes exhibit different gene expression profiles

The data presented here indicate basic differences between hepatocytes containing diploid and polyploid nuclei. Based on the finding that insulin binding inversely correlates with nuclear ploidy, the separation of hepatocytes was performed 15 min after incubation with 10 μM insulin by FC, thereby allowing the separation of hepatocytes with diploid and polyploid nuclei, respectively, without exposure to PI. Gene expression of diploid and polyploid nuclei containing hepatocytes was assessed by microarray analyses with non-sorted cells from the same mouse as control.

**Figure 9:**
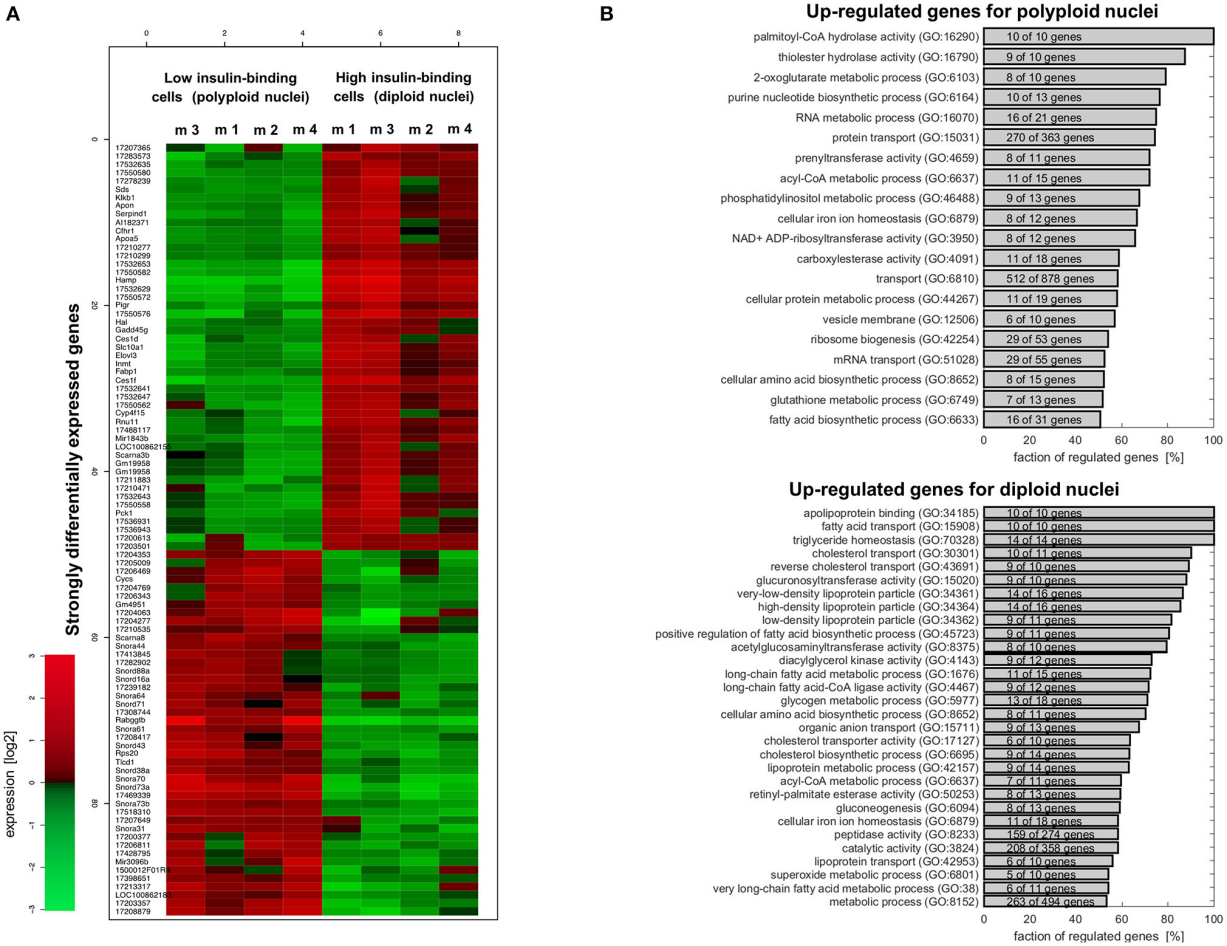
Microarray analysis reveals major differences at the transcriptional level between hepatocytes having diploid or polyploid nuclei. **(A)** Heatmap for differentially regulated genes (*p* < 0.01 and fold-change >2) in insulin high-binding (diploid nuclei) and low-binding (polyploid nuclei) hepatocytes. Supplementary Table 2 provides the list of all differentially expressed genes. **(B)** Functional analysis of the expression differences based on gene ontology categories performed by the Gene Set Regulation Index (Supplementary Material). The figure shows metabolic functions where at least 50% of genes are upregulated in polyploid (upper panel) and diploid (lower panel) hepatocytes. All the GO categories can be found in Supplementary Tables 3, 4. The corresponding figure for the signaling categories are provided as Supplementary Figures 4, 5.

Based on the Gene-set Regulation Index (Bartholomé et al., 2009), around 32% of the genes are differentially expressed in diploid and polyploid nuclei containing hepatocytes (see Supplementary Figure 2), indicating pronounced differences between both entities at the transcriptional level. Among them, 252 genes show a more than 2-fold change, and 1,661 genes an at least 1.5-fold change. The largest positive regulation in diploid hepatocytes (isolated as high insulin binding cells) was found for Rabggtb (up-regulated by a factor of 14.9). In polyploid hepatocytes (isolated as low insulin binding cells), Hamp was up-regulated by a factor of 13.6. The complete list of significantly differentially expressed genes is provided in Supplementary Table 3, including fold-change estimates and *p*-values. Figure 9A shows the subset of genes, with *p* < 0.01 and fold-change larger than a factor of 2 between strictly diploid and polyploid hepatocytes. According to these thresholds, 48 genes were upregulated (red) in diploid hepatocytes, and 45 genes were downregulated (green) in these cells.

Functional analysis of the differentially expressed genes based on gene ontology showed a complex picture suggesting an intricate functional difference between both entities. Supplementary Table 3 shows the gene set regulation index as an estimate of the fraction of differentially expressed genes for all gene ontology categories with more than 10 genes and with more than 50% of genes upregulated in cells with polyploid nuclei. Supplementary Table 4 shows the respective outcomes for categories with more than 50% of genes upregulated in cells with diploid nuclei.

Altered expression could be observed in numerous gene ontology categories related to the metabolism of hepatocytes (shown in Figure 9B). In hepatocytes with polyploid nuclei, most genes associated with RNA-, phosphatidylinositol-, and gluthathione metabolism, with protein- and RNA transport as well as genes involved in biosynthesis of purine nucleotides, ribosomes, amino acids, and fatty acids showed up-regulation. In diploid nuclei containing cells on the other hand, the majority of genes were up-regulated which are involved in glycogen metabolism and gluconeogenesis, in cholesterol- and fatty acid transport, as well as constituents of VLDL, LDL, and HDL lipoproteins.

Genes of signaling categories in the GO annotation also exhibit an intricate regulation between both entities as depicted in Supplementary Figures 4, 5. In cells with polyploid nuclei which were selected due to less insulin binding, more than 50% of genes assigned to NFκB-, RAS-, TNF-, and JAK-STAT signaling are upregulated. Upregulation of these pathways might render hepatocytes with polyploidy nuclei as better survivors compared to hepatocytes with diploid nuclei. In line with this, hepatocytes with diploid nuclei exhibiting enhanced expression of genes “inducing apoptosis by extracellular signals (GO:0008624),” but also enhanced insulin binding and the majority of genes related to JNK-, insulin/IGF1-, IL1-, and WNT signaling as well as negative regulators of MAPK- and BMP signaling are upregulated. However, because gene expression levels provide only an incomplete picture about the abundance of the respective proteins and since the quantitative impact of regulation of pathway compounds on the strength of signaling pathways is unknown, it is difficult to reliably draw concrete conclusion without further experimental investigation. Nevertheless, our observations indicate that both cell entities are characterized by different regulation of pathway constituents and therefore unequal sensitivity for the respective signaling pathways.

## Discussion and summary

Polyploidy of hepatocytes is a known biological phenomenon in most mammals and develops postnatally during liver growth (Styles, 1993). Since a separation of diploid binuclear and pure polyploid cells, respectively, is difficult (Severin et al., 1984), insights into their functional characteristics are still limited.

By combining flow cytometry and HCS we were able to identify, quantitate and characterize different entities of hepatocytes in the liver of mice based on the number of nuclei per cell and their respective ploidy status. The results obtained by flow cytometry directly after hepatocyte isolation were comparable with those obtained by HCS after overnight culture, despite the loss of some hepatocytes due to apoptosis. Apoptotic cells were observed for 2n, 4n or >4n after overnight incubation but significant differences to freshly isolated cells could only be shown for the 2n and 4n populations (Figure 1C). Despite this difference, the fact that all three populations could be found after overnight cultivation and the possibility of separating and quantifying mono and binuclear cells in addition to their polyploidy renders HSC suitable for functional analyses of hepatocytes with different ploidy. Using HSC we found that over 55% of the hepatocytes were binucleated. Around 15% of the cells were mononuclear diploid cells and 26% were binuclear diploid cells. In addition, 15% of the cells were mononuclear with polyploid nuclei, and 24% were binuclear cells with polyploid nuclei (Figure 1F).

Interspecies studies of the ploidy status of liver cells indicate a correlation between high postnatal growth rate associated with increased DNA content, and the species-specific polyploidy level which presumably increases in response to metabolic requirements (Vinogradov et al., 2001). Other studies suggest that liver cell polyploidy closely correlates with postnatal liver growth, while the rate of basal metabolism only correlates with the frequency of binucleated hepatocytes (Anatskaya et al., 1994). Our quantitative analysis of GFP expression after adenoviral infection as an indicator for basal transcriptional turnover in hepatocytes revealed that liver cells with polyploid nuclei express 10–100 times more GFP than diploid cells (Figure 2). These results suggest that in the adult liver, nuclear polyploidy has a strong impact on transcriptional turnover, e.g., on the basal overall gene expression level of hepatocytes (Figure 9 and Supplementary Data). This indicates that nuclear polyploidy may have a stronger impact than the number of nuclei per cell, although we could not directly compare both effects.

The cytochrome P-450 system is central to the metabolism of xenobiotics in the liver. Furthermore, the conversion of fluorescein from non-fluorescent to fluorescent substrates within hepatocytes has been shown to correlate closely with cytochrome activity and albumin production in the cell (Miller, 1983; Nyberg et al., 1993). We utilized the non-fluorescent agent CFDA-SE, which is retained intracellularly once it has been enzymatically converted to fluorescein, as a marker for substrate induced enzymatic activity in the hepatocyte, and correlated this metabolic activity to DNA content of mouse hepatocytes. Flow cytometry performed 10 min after CFDA-SE incubation could clearly distinguish between 2n hepatocytes with high enzymatic activity and 8n cells with lower activity, while binuclear diploid and mononuclear polyploid hepatocytes (4n) could not be distinguished by this method. The bimodally distributed amounts of fluorescein observed in the 4n cells suggest, however, that binuclear and mononuclear diploid liver cells had similar fluorescein conversion activity (Figure 3). Contrary to the data obtained after adenoviral infection, diploid hepatocytes converted much more substrate than polyploid liver cells, suggesting major differences between these cells with respect to basal and substrate-induced metabolism is in line with our functional analysis of the gene expression data.

Maintenance of metabolic homeostasis and metabolic adaptation to nutritional changes are critical for survival. In this context, the liver is of central importance for the maintenance of glucose homeostasis (Moore et al., 2012). Insulin is the primary hormone controlling glucose uptake and release by the liver (Postic et al., 2004). At the hepatocellular level this is mediated by activation of the insulin signaling pathway, initiated by binding of insulin to the membrane-associated IR and followed by the activities of a complex signaling network mediating their metabolic actions (Taniguchi et al., 2006). Interestingly, our analyses again identified two liver cell subtypes with different insulin binding characteristics (Figure 4): mono- and binuclear hepatocytes with diploid nuclei (2n and 2^*^2n) showed increased amounts of insulin binding, whereas mono- and binuclear liver cells with polyploid nuclei (4n and 2^*^4n) exhibited low levels of insulin binding.

Since apoptotic hepatocytes and other cells types are only able to bind insulin to a much lower extent, FITC intensity strongly depends on the selection of cells in the forward vs. side scatter bivariate plot. We established an application-specific automatic separation procedure for discriminating viable hepatocytes from dead hepatocytes and from other cell types present in liver tissues, such as hepatic stellate cells or Kupffer cells as presented in Figure 5. A mixture model accounting for the bimodality was applied to estimate time and dose-dependency of insulin binding (Figure 6). In this way it was possible to accomplish both, a robust analysis which is insensitive to the choice of thresholds, and combining of hundreds of data sets obtained in different cell preparations. Cells with diploid nuclei showed an increased magnitude of insulin binding by a factor of around 16 compared to cells with polyploid nuclei. The kinetic pattern of dose-dependency for insulin was similar (Figure 7).

Since insulin receptor (IR) localization, expression, and sensitivity for insulin stimulation are intricately regulated, there are several possible mechanistic interpretations for the observation. Our model, which is based on ordinary differential equations for the observed kinetics, predicts different numbers of available insulin binding sites between diploid and polyploid hepatocytes (Supplementary Figure 1, Supplementary Table 1), even though experimentally we could not identify a difference in IR expression (Figure 8A) or localization. In addition, differences in the expression of IR splice variants A and B (Mosthaf et al., 1990) could also be excluded (Figure 8B). Therefore, we hypothesize that other mechanisms like differential localization in cellular compartments or different levels of receptor clustering could be a key to explaining the differences between low and high insulin binding liver cells.

The evolutionary benefits raised by the heterogeneity induced by nuclear ploidy are unknown. We can only speculate that the two entities render the liver more robust in stressed situations like detoxification. Moreover, since the liver is a major regulator of insulin degradation and because after insulin release by the pancreas, the blood first passes the liver before systemically circulating through the body, the existence of two entities of hepatocytes with differential responsiveness for insulin might indicate a more robust and/or more efficient modulation capacity for insulin. Since insulin-dependent transporter GLUT4 is not expressed in the liver, and glucose uptake occurs instead via the insulin-independent GLUT2, there seems to be no direct implication for glucose regulation.

A major consequence of polyploidy is an increase in cell volume (Cavalier-Smith, 1978). Therefore, changes in ploidy status result in a change of the ratio of cell surface to cell volume. This in turn may affect metabolic activities, especially those involving signaling pathways and membrane-associated receptor phosphorylation (Weiss et al., 1975). However, since there is no significant difference in the volume of binuclear diploid and mononuclear polyploid hepatocytes (Martin et al., 2002) (Figure 4A), discrepancies observed in insulin binding according to nuclear polyploidy cannot be explained by a difference in cell volume.

Another aspect is that polyploidy of hepatocytes not only correlates with cell size but may also depend on the localization within the hepatic lobule, with periportal hepatocytes being preferentially diploid and pericentral hepatocytes being polyploid (Gandillet et al., 2003; Asahina et al., 2006). The known periportal-pericentral gradient of oxygen, hormones and metabolites as well as the established zonation of metabolic functions (Gebhardt, 1992; Jungermann, 1995), e.g., gluconeogenesis and urea synthesis occurring primarily in the periportal zone and glycolysis and glutamine synthesis being exclusively catalyzed pericentrally, suggest a zonation of the ploidy of liver cells, i.e., the predominant localization of diploid cells in portal areas and polyploid cells in central areas, respectively. We applied insulin *ex vivo* directly into the liver thought the vena porta but could not see a gradient in insulin binding along the periportal-pericentral axis (Supplementary Figure 3). There was no indication that nuclear polyploidy differ along this axis which is in agreement with an earlier study (James et al., 1986) arguing that the metabolic zonation and the ploidy of liver cells are independent biological features.

Given the inverse ploidy patterns in liver and heart, changes in gene expression were mainly associated with a shift from oxidative to anaerobic pathways in polyploid tissues such as the liver (Anatskaya and Vinogradov, 2007). Polyploidy protects among other things against stress-related apoptosis, DNA damage, hypoxia and reactive oxygen species, and increases the metabolic plasticity of cells, thereby promoting the maintenance of their tissue-specific functions and overall survival (Anatskaya and Vinogradov, 2010). In fact, our results obtained after overnight cultivation could corroborate a greater sensitivity for apoptosis in the diploid hepatocytes as compared to the polyploid ones (Figure 1C). Gene expression profiles in microarray analyses of hepatocytes isolated according to their ploidy status but not according to the number of nuclei per cell found no major changes (Lu et al., 2007). By contrast, our microarray analyses were performed with hepatocytes which differed not only in their total ploidy status but also in the number of nuclei per cell, thus separating mononuclear polyploid and binuclear diploid hepatocytes, revealing around 32% differentially expressed genes with expression differences up to 15-fold (Figure 9 and Supplementary Figure 2).

The functional analysis of these genes shows a complex picture. Genes involved in several signaling pathways and metabolic functions have been found to be up-regulated in hepatocytes with polyploid nuclei (Figure 9 and Supplementary Figures 4, 5), while genes involved in other signaling pathways or metabolic functions like fatty acid and glycogen metabolism, ion transport or calcium ion binding were up-regulated in cells with diploid nuclei. This result is in agreement with a higher substrate-induced metabolism in hepatocytes with diploid nuclei compared to cells with polyploid nuclei, which are characterized by a higher basal level of metabolism (Supplementary Tables 3, 4).

Taken together, our analyses show that hepatocytes with diploid and polyploid nuclei have different biological properties. While nuclear polyploidy increases basal protein synthesis and protection against apoptosis, nuclear diploidy correlates with enhanced substrate-induced enzymatic liver cell functions and the capacity to bind insulin. This finding emphasizes the relevance of the cellular diversity found in the liver and suggests major differences in biological functions of the liver which are regulated by insulin: glucose uptake, storage and release, as well as gluconeogenesis.

Due to the existence of polyploid hepatocytes in both periportal and pericentral areas, we suggest the ploidy status of individual hepatocytes to be a further level of biological heterogeneity of liver cells. Although the mechanism leading to the genesis of polyploidy in the hepatocyte is still not understood in detail, the total ploidy status of individual hepatocytes, as well as their nuclear ploidy, adds further levels of biological heterogeneity of liver cells beyond the well-known metabolic zonation, and seems to be critical for the function of the liver parenchyma. Further research should address whether changes in the pattern of polyploidy along the sinusoid could have consequences for the function of the liver parenchyma and may influence liver diseases.

## Author contributions

CK wrote parts of the manuscript, statistically analyzed the data and established the kinetic model. JT supervised and designed the project. MF performed the data obtained by HSC. SM performed the animal preparations, AW and PB performed all the experimental data. MB wrote parts of the manuscript, analyzed the experimental obtained data and supervised and designed the project.

### Conflict of interest statement

The authors declare that the research was conducted in the absence of any commercial or financial relationships that could be construed as a potential conflict of interest. The SH and handling Editor declared their shared affiliation.
