# Supplementary material for: Hepatocyte Ploidy Is a Diversity Factor for Liver Homeostasis
Source: Front Physiol. 2017 Oct 31;8:862. doi: 10.3389/fphys.2017.00862 (PMC5671579; doi:10.3389/fphys.2017.00862)

Supplementary Material

Hepatocyte ploidy is a diversity factor for liver homeostasis

Clemens Kreutz^1,2*^, Sabine MacNelly^4^, Marie Follo^5^, Astrid Wäldin^4^, Petra Binninger-Lacour^4^, Jens Timmer^1,3^, María Matilde Bartolomé Rodríguez^4^

^1^Institute of Physics, Herman Herder Str. 3, University of Freiburg, 79104 Freiburg, Germany

^2^Center for Systems Biology, Habsburger Str. 49, University of Freiburg, 79104 Freiburg, Germany

^3^BIOSS, Centre for Biological Signalling Studies, Herman Herder Str. 3, University of Freiburg, 79104, Freiburg, Germany

^4^Clinic for Internal Medicine II/ Molecular Biology, Hugstetter Str. 55, Medical Center - University of Freiburg, Faculty of Medicine, University of Freiburg, 79106 Freiburg, Germany

^5^Clinic for Internal Medicine I/Lighthouse Core Facility, Medical Center - University of Freiburg, Faculty of Medicine, University of Freiburg, Germany.

*** Correspondence:** Clemens Kreutz: ckreutz@fdm.uni-freiburg.de

# Analysis of the insulin binding kinetics by an ordinary differential equation based mathematical model

The cell-cell variability of insulin binding within both entities of hepatocytes as given by the width of the FITC histograms measured by flow cytometry were almost independent of time and dose of insulin treatment. Similarly, the relative numbers of cells belonging to the two cell entities were independent of insulin treatment.

In contrast, the average levels of insulin binding in both cell entities show a strong time- and dose dependency as indicated in Figure 7 in the main text. This behavior has been analysed by a basic kinetic model in order to predict the main mechanistic differences between the two entities of hepatocytes. The model describes the time dependency of the concentration of insulin *I(t)*, receptors *R(t)* and insulin receptor complexes *C(t)*. According to the chemical mass action law for complex formation, it consists of the following three *ordinary differential equations (ODEs)*

$$\dot{I}=- k_{a}I R+ k_{d}C$$

$$\dot{R}=- k_{a}I R+ k_{d}C$$

$$\dot{C}= k_{a}I R- k_{d}C$$

describing the dynamics of the concentration of three compounds in a single entity. In each equation, the temporal change is proportional to the concentrations of the reactants. The dynamics *I(t)*, *R(t)* and *C(t)* are calculated by integration of the three differential equations. *
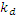
k_a_*
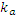
denotes the association rate and *
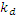
k_d_*
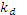
the dissociation rate. A third parameter was the number of receptors, or in a more general sense, the number of binding sites *R(t=0).*

The three parameters *
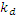
k_a_*, *
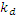
k_d_* and *R(t=0)* have been checked for being different in both groups of cells. For this purpose, three additional fold-factor parameters for the difference of
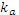
*
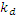
k_a_ ,
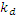
k_d_* and *R(t=0)* were introduced. The total of six parameters were estimated by maximum likelihood ^1^, with confidence intervals derived by the profile likelihood methodology ^2,3^ (Supplementary Table S1).

In order to test statistically whether the parameters are different for both cell entities, likelihood ratio tests ^4^ were performed indicating a significant increase in the number of binding sites (p= 4.7e-6) in one of the hepatocyte entities by a factor of 16.2 compared to the other entity. The 95% confidence interval for this fold-change is [10.1, 25.8], which means that the observed increase is more than a factor of ten. Although the differences were not significant for the association rate *
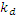
k_a_* (p=0.74), nor for the dissociation rate *
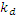
k_d_* (p=0.52), a slightly decreased dissociation rate in diploid hepatocytes was weakly indicated.

Since flow cytometry provides only relative data with multiplicative errors, the data were fitted on the logarithmic scale. The following observation function

*log( y(t) ) = log( b_0_ + b_1_ C(t) ) + ε*

has been used to fit the dynamic model. *y(t)* denotes the measurements at time point *t*, *b_0_* is an offset parameter, *b_1_* a scaling parameter and *C(t)* denotes the concentration of bound insulin. The variance *σ^2^* of the measurement errors *ε ~ N(0, σ^2^)* has been calculated during pre-processing of the data, i.e. was derived from experimental replicates.

# Quantitative analysis of data obtained by Affymetrix microarrays

Experimental data obtained by Affymetrix GeneChips were preprocessed using the Robust Multi-Array Analysis (RMA)^5^. A linear model was used to test for significantly regulated genes between the two entities of hepatocytes, as well as for the estimation of the fold-changes and adjusting for differences between different preparations. Supplemental Figure S2 shows the distribution of the p-values assessing the significance of expression differences between the two cell types. The histogram exhibits a clear shift towards zero, indicating global expression differences. The Gene Set Regulation Index (GSRI) ^6^ as an estimate of the number of regulated genes is 31.9%, indicating that almost one third of the genes are significantly regulated. A list of the significantly regulated genes is provided in Supplemental Table S2.

The gene-set regulation index was applied to estimate the percentage of regulated genes within groups of genes. The up- and downregulated gene ontology (GO) categories are provided in Supplementary Table S3 for hepatocytes with polyploid nuclei (low insulin binding cells) and Supplementary Table S4 for hepatocytes with diploid nuclei (high insulin binding cells).

# Supplementary references

1. Kreutz, C., Raue, A. & Timmer, J. in *Multiple Shooting and Time Domain Decomposition Methods* (ed. Carraro T., Geiger M., Körkel S.) 355–375 (2015). doi:10.1007/978-3-319-23321-5_14

2. Meeker, W.Q. & Escobar, L. A. Teaching about Approximate Confidence Regions Based on Maximum Likelihood Estimation. *Am. Stat.* **49,** 48–53 (1995).

3. Raue, A. *et al.* Structural and practical identifiability analysis of partially observed dynamical models by exploiting the profile likelihood. *Bioinformatics* **25,** 1923–1929 (2009).

4. Cox, D. *Theoretical Statistics*. (Chapman & Hall, 1994).

5. Irizarry, R. A. *et al.* Summaries of Affymetrix GeneChip probe level data. *Nucleic Acids Res.* **31,** e15 (2003).

6. Bartholomé, K., Kreutz, C. & Timmer, J. Estimation of gene induction enables a relevance-based ranking of gene sets. *J. Comput. Biol.* **16,** 959–67 (2009).

# Supplementary Tables


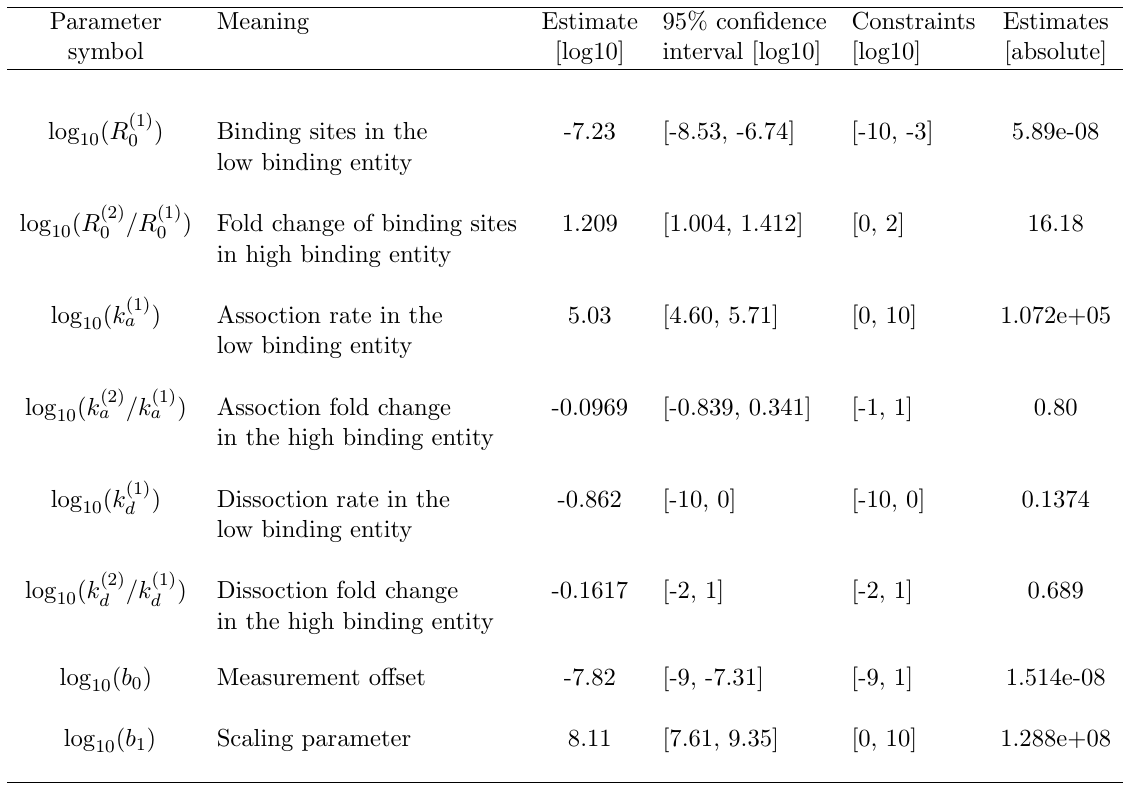


**Supplementary Table S1: Estimated parameters for the dynamic model.** Estimation has been performed on the log10-scale for numerical reasons and to account for the fact that the parameters are strictly positive. 95% confidence intervals have been calculated based on the profile likelihood. The order of the denoted parameters corresponds to panels (A)-(H) in Supplementary Figure S1. The most relevant outcome is the estimated difference for the number of binding sites. The estimated value 1.209 on the log10-scale corresponds to a factor of 16.18 on the absolute scale. The respective confidence interval [1.004, 1.412] is [10.09, 25.82] on the absolute scale. The fold-factors for the dissociation rate 0.80 and for the dissection rate 0.689 are close to 1 on the absolute scale and as previously mentioned not significantly different from a factor of 1. In this table, this is indicated by the confidence intervals on the log-scale which cover a log10-value of zero.

[SupplementaryTable S2.xlsx] (too large to be included in this document)

**Supplementary Table S2. List of differentially regulated genes in cells with high insulin affinity (hepatocytes containing diploid nuclei).** The fold-factors were calculated based on a linear model on the logarithmic scale. The linear model also provides p-values for the fold-changes based on the t-statistic. The last column indicates the direction of the regulation.

[SupplementaryTable S3.xlsx] (too large to be included in this document)

Supplementary Table S3. List of upregulated GO categories in cells with low insulin affinity (hepatocytes with polyploid nuclei).

All categories with an estimated fraction of at least 50% genes (GSRI>0.5) are shown. The first two columns indicate the GO category. The third column shows the number of genes which were assigned to the respective category. The gene set regulation index is the estimated proportion of regulated genes in the category. Furthermore, the absolute number of regulated genes is provided in the fifth column. In addition, the median p-value, the geometric mean of the p-values and the average fold-change are given. The fold-changes have been averaged at the log2-scale, i.e. -1 would correspond to an average downregulation by a factor of ½ in high-binding cells, which is equivalent to upregulation in the low-binding cells. The last column is the p-value obtained by a Kolmogorov-Smirnov test for assessing whether the p-values for the fold-changes are uniformly distributed. The smaller this p-value, the more significant the regulation is in the respective GO category.

[SupplementaryTable S4.xlsx] (too large to be included in this document)

Supplementary Table S4. List of upregulated GO categories in cells with high insulin affinity (hepatocytes with diploid nuclei).

All categories with an estimated fraction of at least 50% genes (GSRI>0.5) are shown. The first two columns indicate the GO category. The third column shows the number of genes which were assigned to the respective category. The gene set regulation index is the estimated proportion of regulated genes in the category. Furthermore, the absolute number of regulated genes is provided in the fifth column. In addition, the median p-value, the geometric mean of the p-values and the average fold-change are given. The fold-changes have been averaged at the log2-scale, i.e. +1 would correspond to an average upregulation by a factor 2. The last column is the p-value obtained by a Kolmogorov-Smirnov test for assessing whether the p-values for the fold-changes are uniformly distributed. The smaller this p-value, the more significant the regulation is in the respective GO category.


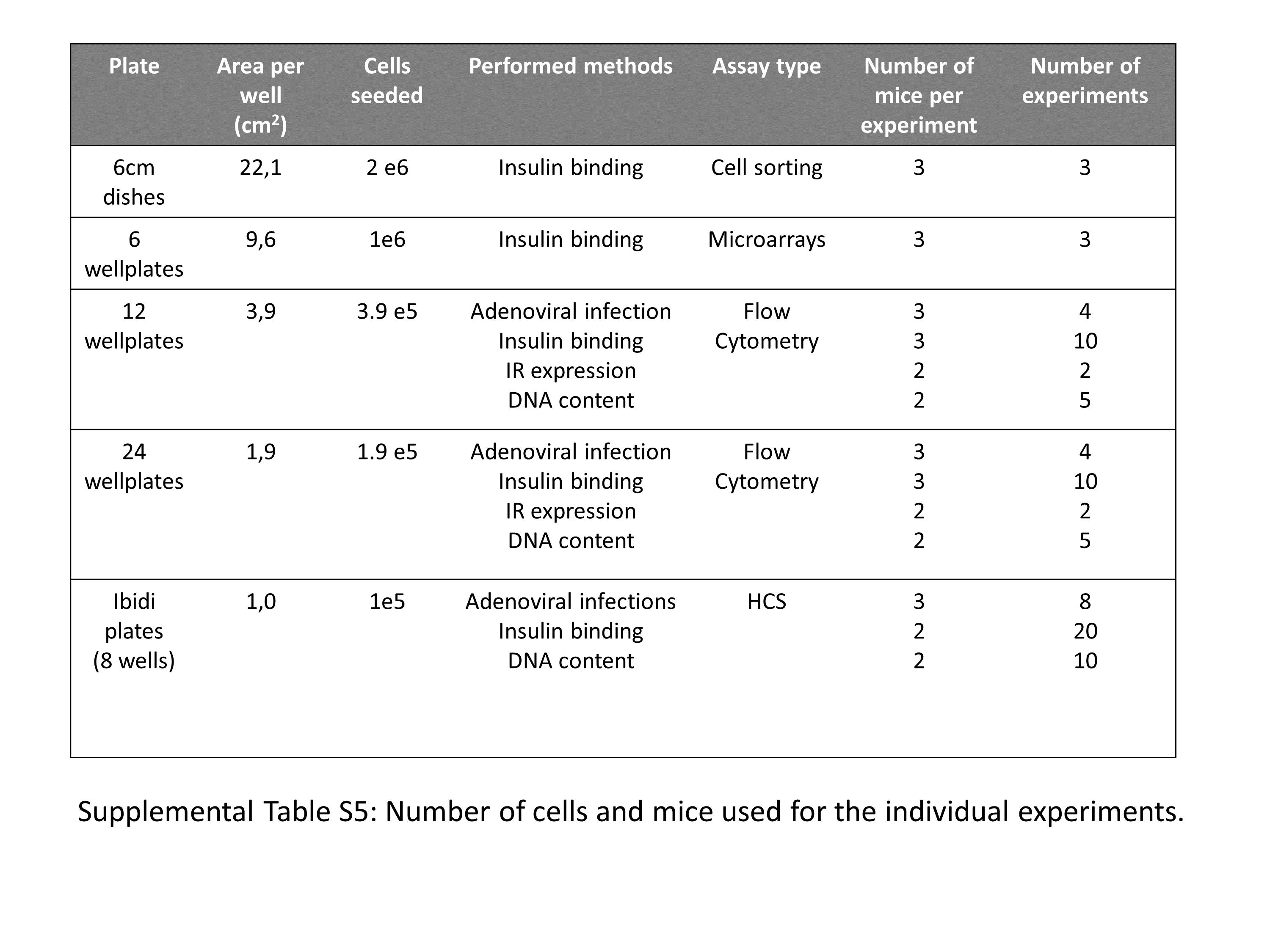

Supplement: Supplementary file 1 [file DataSheet1.DOCX]
