# Supplementary material for: Hepatocyte Ploidy Is a Diversity Factor for Liver Homeostasis
Source: Front Physiol. 2017 Oct 31;8:862. doi: 10.3389/fphys.2017.00862 (PMC5671579; doi:10.3389/fphys.2017.00862)
Supplement: Supplementary file 5 [file Image1.pdf]

Supplemental Figure S1

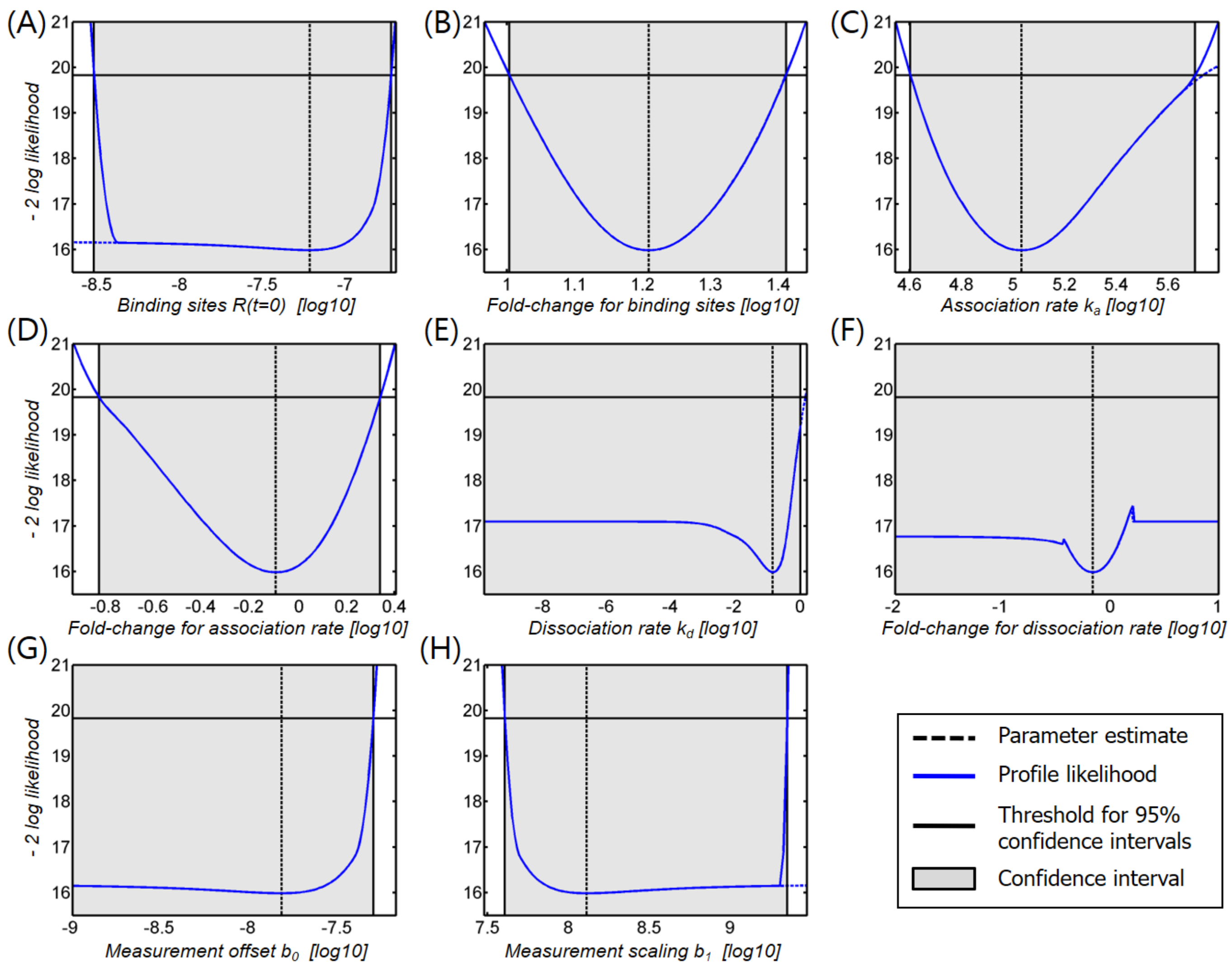

Supplemental Figure S1: The profile likelihood (blue lines) indicates the range of a parameter in statistical agreement with the data.

The profile likelihood has been used to derive confidence intervals (parameter ranged indicated by the gray background) for the estimated parameter. Confidence intervals are given by the intersection points of the profile likelihood (blue lines) with the 95% threshold (black horizontal lines). Roughly said, parameters with a clearly pronounced minimum can be estimated based on the given data. The receptor number in the first cell type, i.e. in the entity with low insulin binding, is not well identifiable (panel (A)) since flow cytometry only provides relative data. Nevertheless, the fold change of the binding sites $log_{10}(R_0^{(2)} / R_0^{(1)})$ is identifiable (B), and its respective confidence interval shows that the second entity has more than one order of magnitude more receptors. According to panel (C), the association rate $log_{10}(k_a^{(1)})$ in the first entity is identified in the range $k_a^{(1)} = [10^{4.6}, 10^{5.7}]$. Because the confidence intervals of the fold-changes of the association rate (D) and of the dissociation rate (F) contain zero on the log10-scale, these parameters are not significantly different between the two entities. The dissociation rate in the low-binding entity (panel (E)) is practically non-identifiable towards the small values. This means that the measurements could also be described by a model without dissociation. The profile likelihood which would be obtained for the corresponding issue without any constraint of parameter space is plotted as a dashed colored line. These lines are visible in panels (A), (C), (D), and (H) and indicate the sensitivity of the profile likelihood and of the confidence intervals on the chosen upper and lower bounds for the parameters.

Without regulation, the p-values would be uniformly distributed between 0 and 1 and the histogram would be flat. In contrast, the observed shift towards zero indicates a global expression difference. The magnitude of the shift, i.e. the area highlighted in red, can be used to estimate the proportion of regulated genes which is the basis of the *Gene-set Regulation Index (GSRI)*. This has been performed for different functionally related subsets of genes according to the gene ontology (GO) annotation. In this way the regulated biological processes could be identified. For the whole measured transcriptome, the GSRI indicates that around 32% of the genes are differentially expressed between hepatocytes from the two entities with a difference in their ability to bind insulin.
