# Supplementary material for: Hepatocyte Ploidy Is a Diversity Factor for Liver Homeostasis
Source: Front Physiol. 2017 Oct 31;8:862. doi: 10.3389/fphys.2017.00862 (PMC5671579; doi:10.3389/fphys.2017.00862)
Supplement: Supplementary file 6 [file Image2.PDF]

Supplemental Figure S2

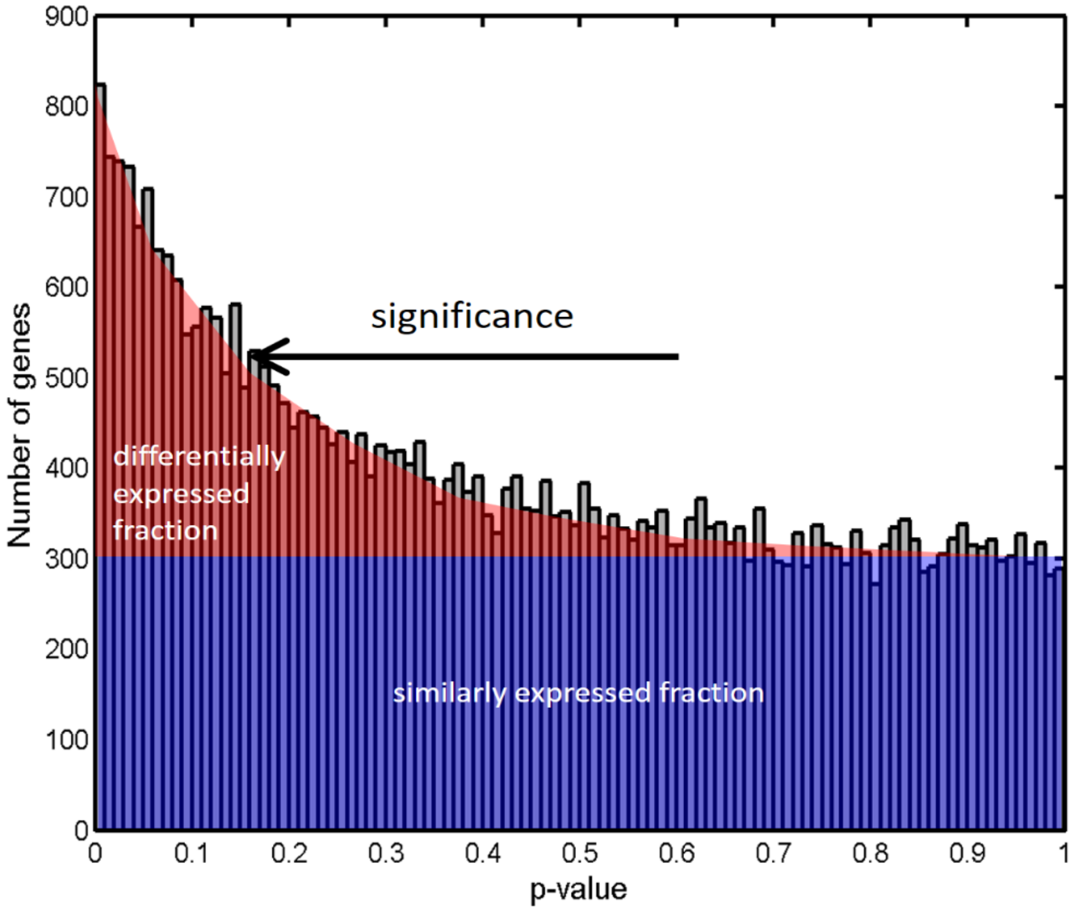

Supplemental Figure S2: Histogram of the p-values (underlying gray bars) for expression differences between the two groups of hepatocytes. The magnitude of the shift towards zero can be utilized to estimate the fraction of regulated genes.

Supplemental Figure S3

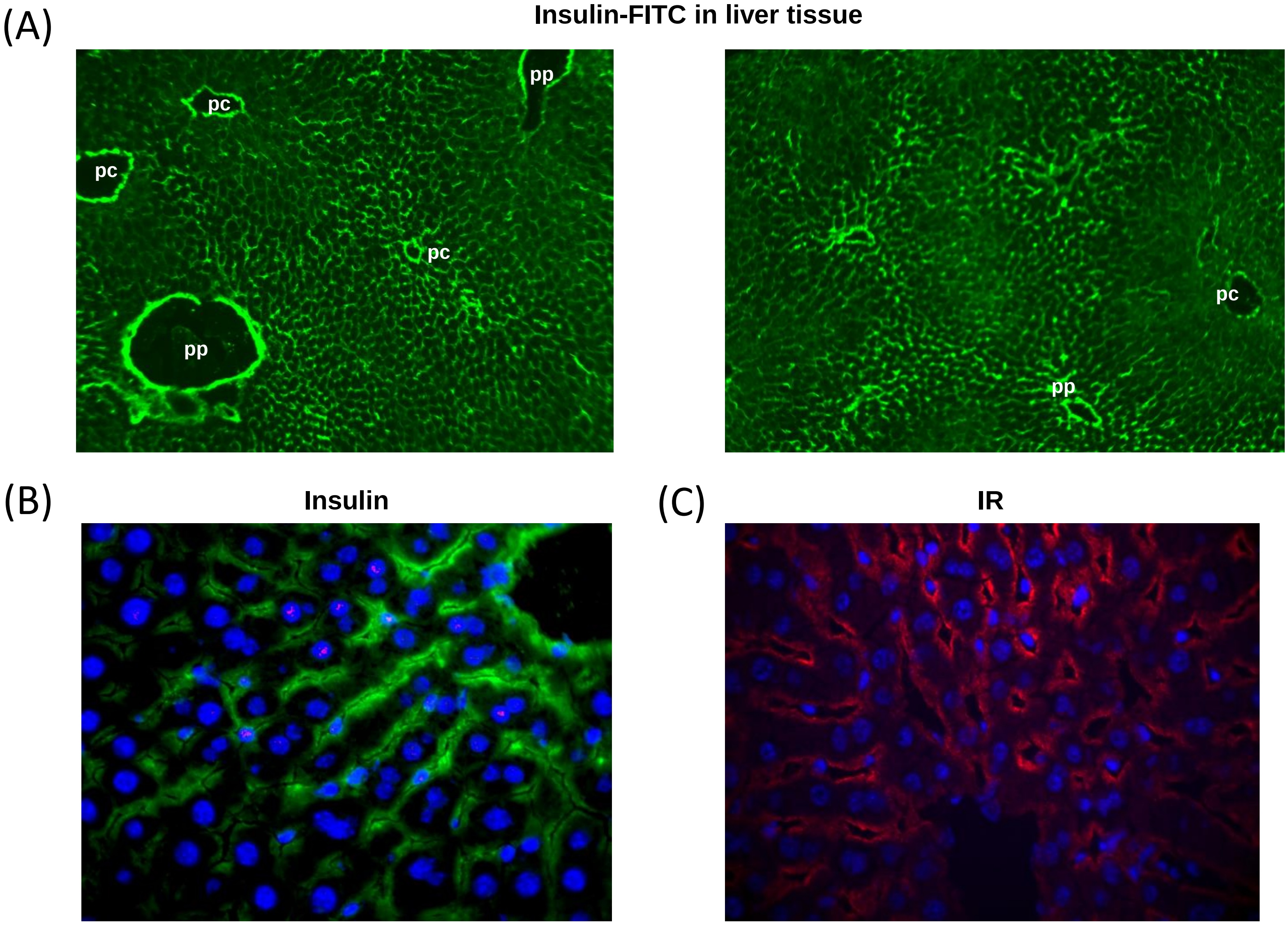

Supplemental Figure S3: Spatial distribution of insulin binding in the liver tissue

(A) Insulin-FITC binding to hepatocyte in the liver tissue after 10 min liver perfusion with insulin-FITC in FBS. There are no spatial gradients between periportal (pp) and pericentral (pc) veins. (B) Insulin binding (green) in liver at higher magnification factor. (C) Insulin receptor expression (red color) in liver tissue. The following antibodies were uses: a-Insulin Receptor, #sc-711 ß-subunit (rabbit, polyclonal) from Santa Cruz, and #sc-0506 a-Rabbit IgG-TR (goat, polyclonal) from Santa Cruz.

Supplemental Figure S4

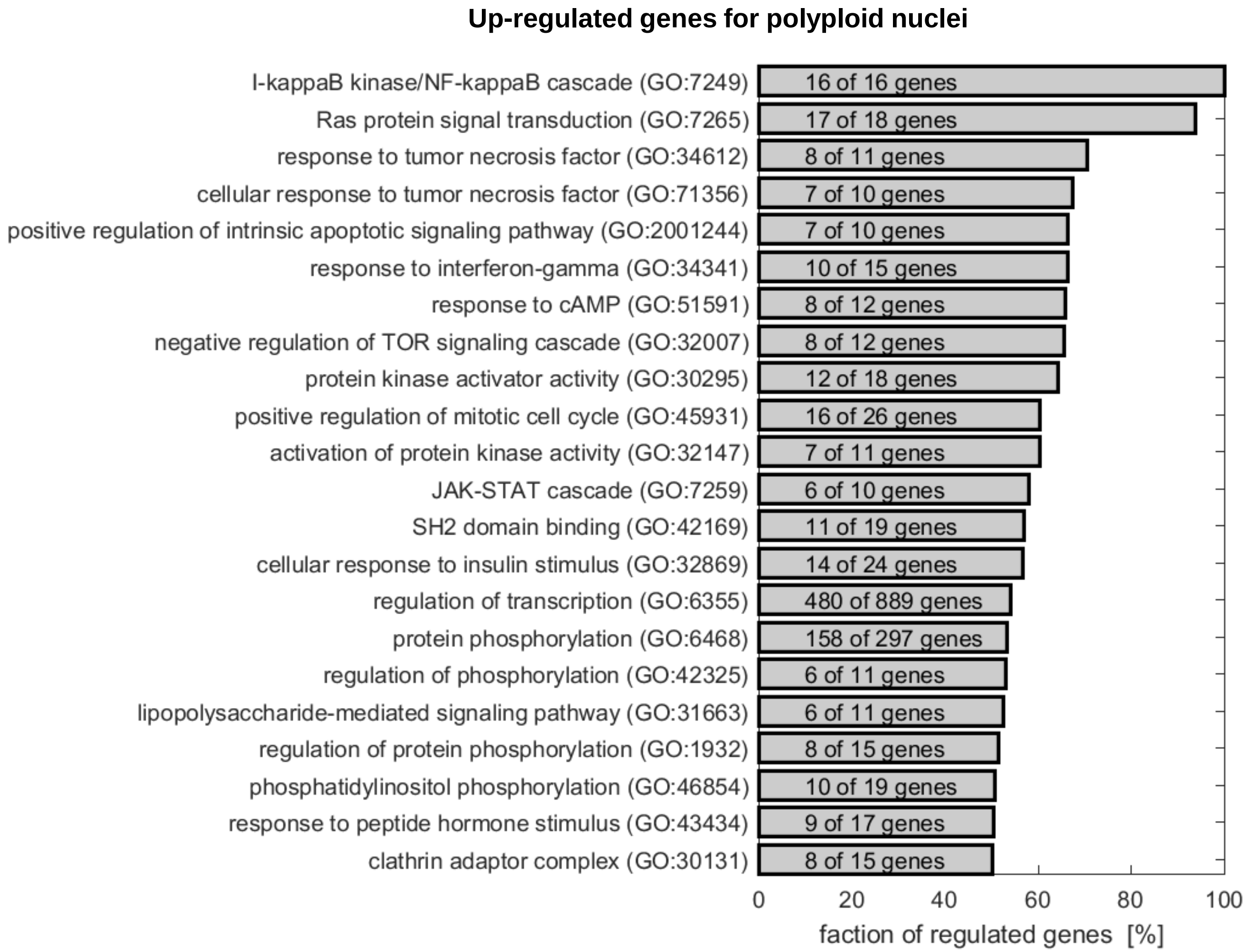

Supplemental Figure S4: Signalling gene ontology categories with more than 50% upregulated genes in hepatocytes with polyploid nuclei.

Supplemental Figure S5

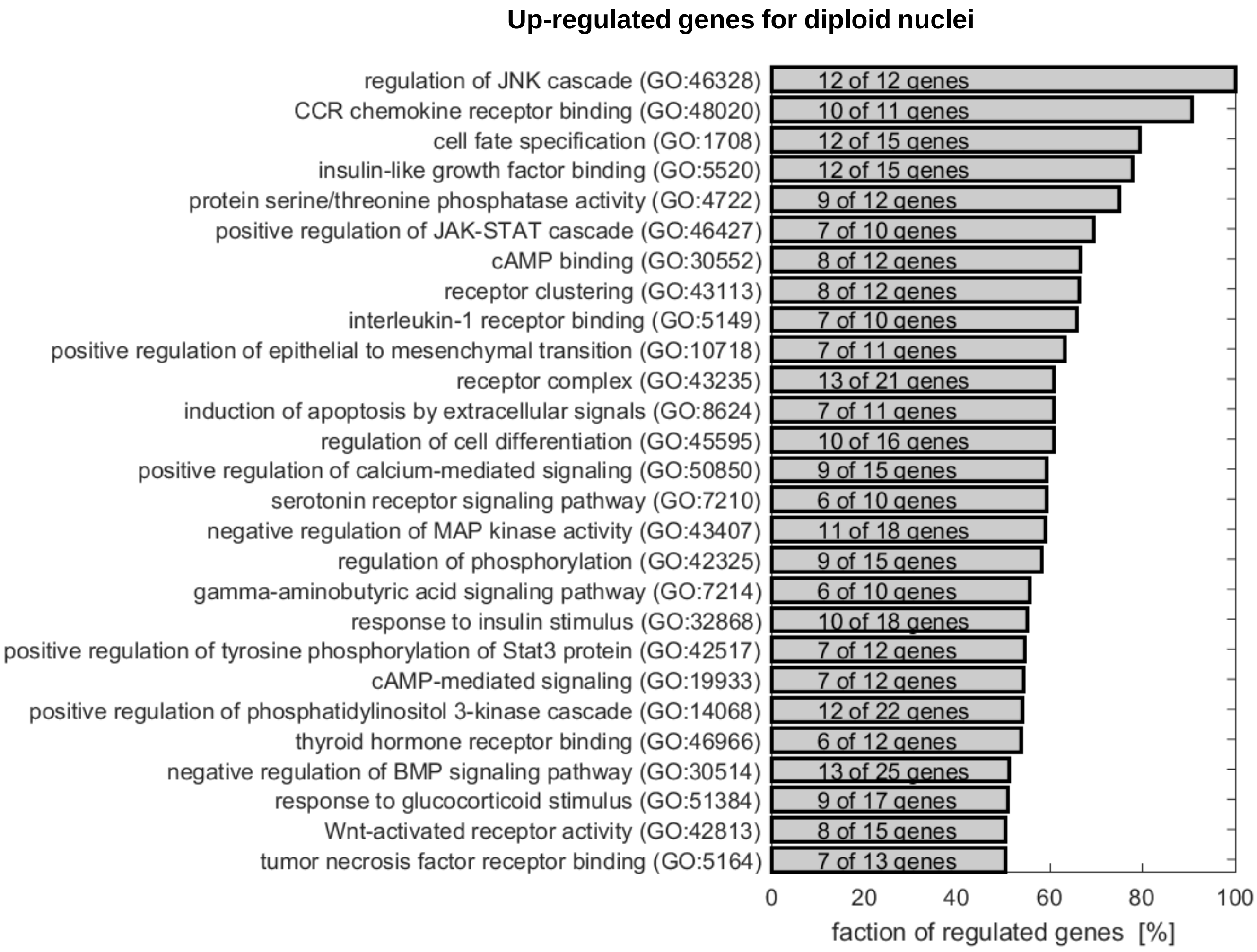

Supplemental Figure S5: Signalling gene ontology categories with more than 50% upregulated genes in hepatocytes with diploid nuclei.
